# Active enterohepatic cycling is not required for the choleretic actions of 24-norUrsodeoxycholic acid in mice

**DOI:** 10.1172/jci.insight.149360

**Published:** 2023-03-22

**Authors:** Jennifer K. Truong, Jianing Li, Qin Li, Kimberly Pachura, Anuradha Rao, Sanjeev Gumber, Claudia Daniela Fuchs, Andrew P. Feranchak, Saul J. Karpen, Michael Trauner, Paul A. Dawson

**Affiliations:** 1 Department of Pediatrics, Division of Pediatric Gastroenterology, Hepatology and Nutrition, Emory University School of Medicine, Children’s Healthcare of Atlanta, Atlanta, Georgia, USA.; 2 Department of Pediatrics, University of Pittsburgh Medical Center Children’s Hospital of Pittsburgh, Pittsburgh, Pennsylvania, USA.; 3 Division of Pathology and Laboratory Medicine, Yerkes National Research Center, Emory University School of Medicine, Atlanta, Georgia, USA.; 4 Hans Popper Laboratory of Molecular Hepatology, Division of Gastroenterology and Hepatology, Department of Internal Medicine III, Medical University of Vienna, Vienna, Austria.

**Keywords:** Gastroenterology, Hepatology, Drug therapy, Toxins/drugs/xenobiotics, Transport

## Abstract

The pronounced choleretic properties of 24-norUrsodeoxycholic acid (norUDCA) to induce bicarbonate-rich bile secretion have been attributed to its ability to undergo cholehepatic shunting. The goal of this study was to identify the mechanisms underlying the choleretic actions of norUDCA and the role of the bile acid transporters. Here, we show that the apical sodium-dependent bile acid transporter (ASBT), organic solute transporter-α (OSTα), and organic anion transporting polypeptide 1a/1b (OATP1a/1b) transporters are dispensable for the norUDCA stimulation of bile flow and biliary bicarbonate secretion. Chloride channels in biliary epithelial cells provide the driving force for biliary secretion. In mouse large cholangiocytes, norUDCA potently stimulated chloride currents that were blocked by siRNA silencing and pharmacological inhibition of calcium-activated chloride channel transmembrane member 16A (TMEM16A) but unaffected by ASBT inhibition. In agreement, blocking intestinal bile acid reabsorption by coadministration of an ASBT inhibitor or bile acid sequestrant did not impact norUDCA stimulation of bile flow in WT mice. The results indicate that these major bile acid transporters are not directly involved in the absorption, cholehepatic shunting, or choleretic actions of norUDCA. Additionally, the findings support further investigation of the therapeutic synergy between norUDCA and ASBT inhibitors or bile acid sequestrants for cholestatic liver disease.

## Graphical abstract

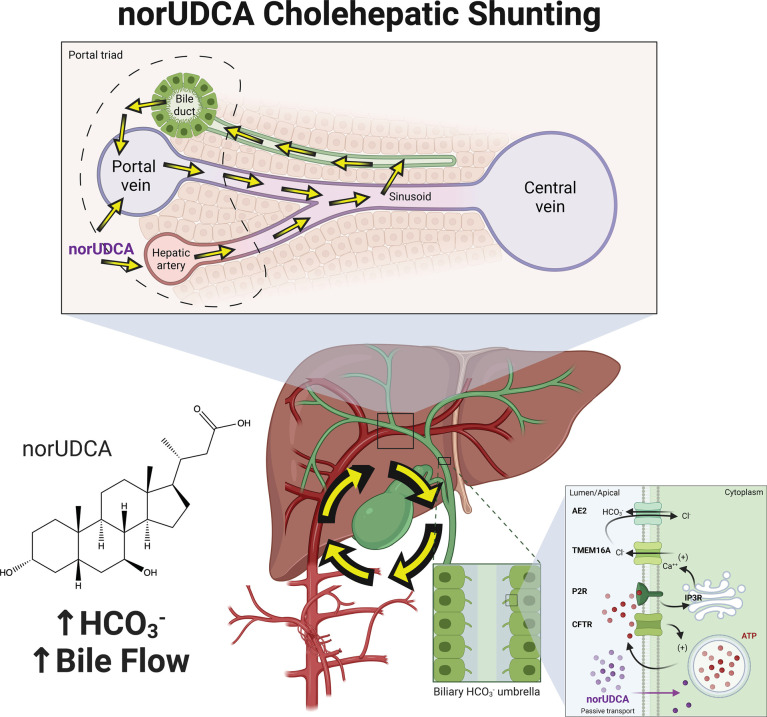

## Introduction

24-norUrsodeoxycholic acid (norUDCA; norucholic acid) is a synthetic C-23 side chain–shortened analog of the hydrophilic native bile acid ursodeoxycholic acid (UDCA) and is resistant to side chain conjugation with glycine or taurine (1). The pharmacological properties and physiological actions of norUDCA make it a therapeutic candidate for a variety of cholestatic liver diseases (1, 2). In preclinical studies, oral administration of norUDCA reduced liver injury and biliary fibrosis in bile duct ligated mice and in (MDR2) *Abcb4^–/–^* mice, whereas administration of UDCA aggravated liver and bile duct injury (3, 4). In those models, norUDCA induced detoxification and renal elimination of bile acids and exhibited antiproliferative, antifibrotic, and antiinflammatory properties (3–7). In Phase 2 clinical trials, administration of norUDCA for 12 weeks reduced serum alkaline phosphatase (ALP) and other liver enzyme markers of cholestasis in patients with primary sclerosing cholangitis (PSC) (8), and it reduced serum alanine aminotransferase (ALT) in patients with nonalcoholic fatty liver disease (9).

The mechanisms of action that mediate the beneficial effects of norUDCA remain the subject of ongoing study (10, 11) but most likely include stimulation of bicarbonate secretion to maintain the protective biliary bicarbonate umbrella (12). Administration of side chain–shortened dihydroxy bile acids such as norUDCA stimulate bicarbonate-rich bile flow, far exceeding that reported for any natural bile acid and more than what can be explained by their osmotic effects (1, 13). The superior capacity of norUDCA versus UDCA to induce a hypercholeresis has been attributed to norUDCA’s ability to evade side chain conjugation (amidation) to glycine or taurine and undergo cholehepatic shunting. In the original pathway proposed over 30 years ago by Hofmann (1, 14), unconjugated norUDCA is secreted by hepatocytes into bile and absorbed in protonated form by cholangiocytes lining the biliary tract, thereby generating a bicarbonate ion from biliary CO_2_. norUDCA then crosses the biliary epithelium and enters the periductular capillary plexus, which drains into the portal vein (or directly into the hepatic sinusoids), delivering norUDCA for reuptake by hepatocytes, secretion into bile, and additional rounds of cholehepatic shunting. Later, it was discovered that apical membrane cyclic AMP (cAMP) and Ca^2+^-activated Cl^–^ channels in cholangiocytes play critical roles in promoting bicarbonate-secretion by the biliary epithelium via coupling to the chloride/bicarbonate anion exchanger (AE2; *SLC4A2*) (15, 16). For example, secretin acting via its basolateral membrane receptor increases intracellular cAMP and stimulates cystic fibrosis transmembrane conductance regulator–mediated (CFTR-mediated) Cl^–^ secretion (17, 18). In addition to CFTR, the Ca^2+^-activated Cl^–^ channel transmembrane member 16A (TMEM16A; also called anoctamin-1 [ANO1]) is expressed by cholangiocytes and plays a particularly important role in regulating biliary anion efflux under basal conditions and in response to stimuli such as nucleotides and bile acids (19, 20). In those studies, activation of TMEM16A by the conjugated therapeutic bile acid tauroursodeoxycholic acid (TUDCA) was dependent upon its apical sodium-dependent bile acid transporter–mediated (ASBT-mediated) uptake into the cell (19). In contrast to native C-24 bile acids, the interaction of side chain–shortened C-23 bile acids such as norUDCA with these mechanisms of biliary secretion remains largely unexplored.

Unmodified norUDCA that escapes absorption in the biliary tract travels along with other biliary constituents into the small intestine, where it is reabsorbed and carried in the enterohepatic circulation back to the liver for reuptake and secretion into bile. In this fashion, norUDCA can undergo multiple rounds of cholehepatic shunting or a combination of enterohepatic cycling and cholehepatic shunting until it is ultimately converted to a more polar metabolite by hepatic phase 1 or phase 2 metabolism (primarily phase 2 glucuronidation) and excreted in the urine or feces (1, 14, 21, 22). The physicochemical and permeability properties of norUDCA include a molecular weight below 500 daltons, a low number of hydrogen bond donors/acceptors, a low octanol-water partition coefficient, and an appreciable intestinal permeability (Supplemental Table 1; supplemental material available online with this article; https://doi.org/10.1172/jci.insight.149360DS1), all of which are generally consistent with a role for passive diffusion in norUDCA’s absorption and cell membrane permeation (23). However, carrier-mediated cellular uptake and export mechanisms also play prominent roles in the absorption and disposition of many drugs and endobiotics (24–26), and the contribution of bile acid and organic anion transporters to the absorption and cholehepatic shunting of norUDCA has not been fully explored (27). In this study, we used mouse and cell-based models to identify the mechanisms by which norUDCA potently stimulates biliary secretion and to determine if major bile acid transporters, including the ASBT and organic solute transporter α-β (OSTα-OSTβ) and an active enterohepatic circulation, are required for norUDCA’s cholehepatic shunting and hypercholeretic effects.

## Results

To determine if the major bile acid transporter ASBT and an active enterohepatic circulation of bile acids are required for the bicarbonate-rich choleresis induced by norUDCA, we examined bile flow and biliary bicarbonate output in background strain-matched WT and *Asbt^–/–^* mice fed chow or chow plus 0.5% norUDCA for 7 days. The experimental scheme and morphological response to norUDCA administration are shown in Supplemental Figure 2. Administration of norUDCA to WT and *Asbt^–/–^* mice for 7 days tended to reduce body weight (Supplemental Figure 2, B and C) but did not affect small intestinal length or weight, colon length or weight, or kidney weight (data not shown). The liver weight and liver weight/body weight ratio were increased in both genotypes with norUDCA treatment (Supplemental Figure 2, D and E). However, the histology was assessed to be normal, and analysis of H&E-stained liver sections revealed no apparent histological differences between the genotypes or treatment groups (Supplemental Figure 2F). Plasma chemistries were not significantly different between the chow and norUDCA-fed groups for both genotypes (Supplemental Table 2).

**Figure 1:**
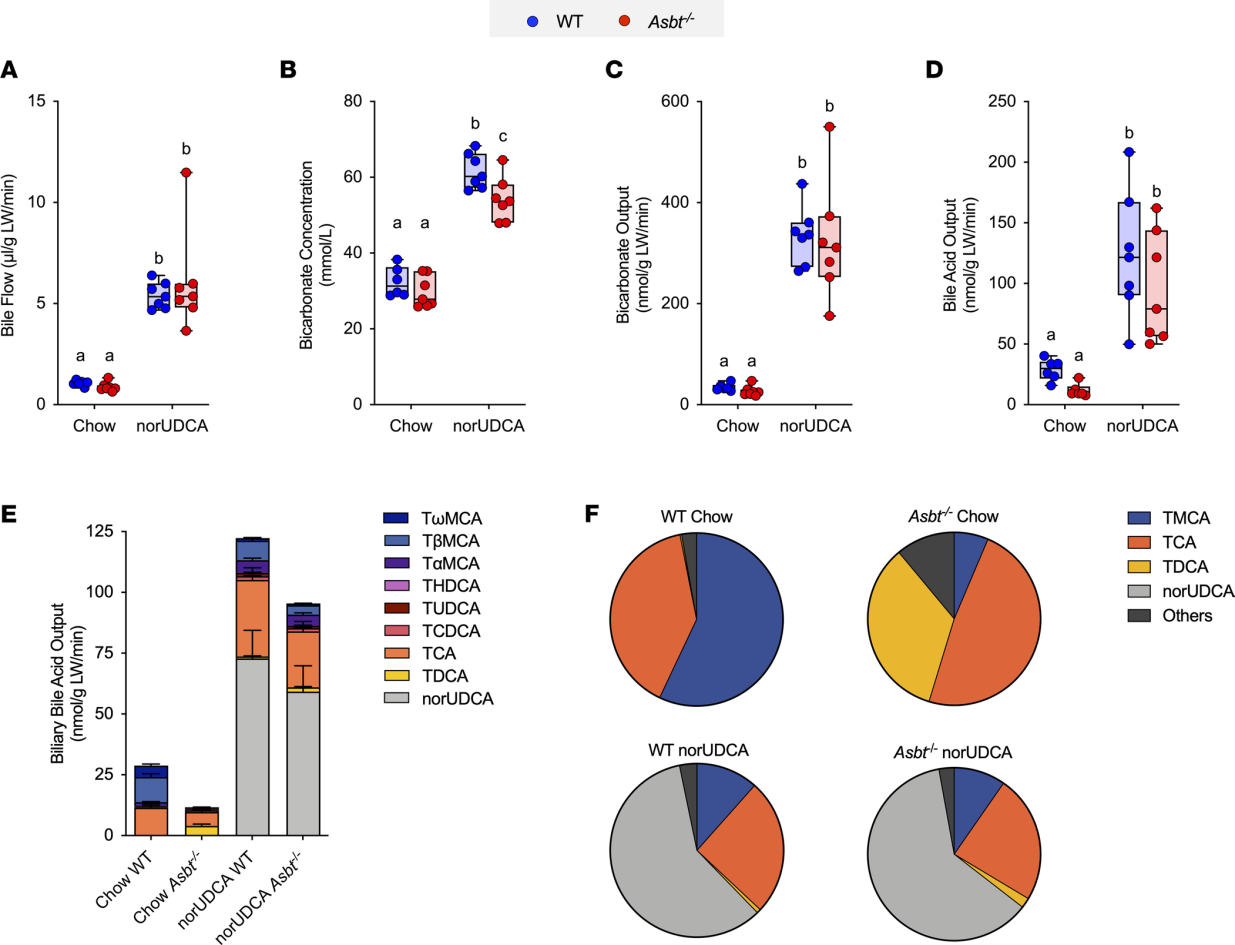
norUDCA treatment increases bile flow and biliary bicarbonate and solute output in WT and *Asbt^–/–^* mice. (**A**) Bile flow. (**B**) Biliary bicarbonate concentration. (**C**) Bicarbonate output. (**D**) Biliary bile acid output. (**E**) Biliary bile acid species output (mean ± SEM). (**F**) Biliary bile acid composition expressed as pie charts. Unless indicated, median values (line), interquartile range (boxes), and minimum to maximum values (whiskers) are shown; *n* = 6–7 mice per group. For stacked bar graph, mean ± SD is shown. The data were evaluated for statistically significant differences using an ordinary 2-way ANOVA with a Tukey’s multiple-comparison test. Distinct lowercase letters indicate significant differences between groups (*P* < 0.05).

The effect of norUDCA administration on bile flow and biliary solute output is shown in Figure 1 and is summarized in Table 1. On the rodent chow diet, bile flow, bicarbonate concentration, biliary bicarbonate output, and bile pH were similar in WT and *Asbt^–/–^* mice. In agreement with a block in ileal active reabsorption of bile acids, the concentration and biliary output of bile acids were reduced by more than 50% in chow-fed *Asbt^–/–^* versus WT mice (Figure 1, D and E). As compared with chow-fed mice, administration of norUDCA increased the bile flow rate by 5- to 6-fold, biliary bicarbonate concentration by 2-fold, and bicarbonate output more than 10-fold in both WT and *Asbt^–/–^* mice (Figure 1, A–C, and Table 1). norUDCA feeding also increased bile acid output by approximately 4-fold and 8-fold in WT and *Asbt^–/–^* mice, respectively (Figure 1, D and E). Since the ability of norUDCA to stimulate a bicarbonate-rich choleresis is thought to be secondary to its potential for cholehepatic shunting and enrichment in bile, biliary bile acid composition was determined for chow and norUDCA-treated WT and *Asbt^–/–^* mice. The output and relative proportion of each bile acid species are shown (Figure 1, E and F). As compared with chow-fed WT mice, *Asbt^–/–^* mice had a more hydrophobic bile acid composition, with reduced relative amounts of 6-hydroxylated bile acid species such as tauro-β-muricholic acid (TβMCA) and increased amounts of taurocholic acid (TCA) and its gut microbiota-derived product taurodeoxycholic acid (TDCA). Following administration of norUDCA, the biliary bile acid composition became more hydrophilic in *Asbt*^–/–^ mice and remarkably like WT mice, with norUDCA accounting for approximately 60% of the total biliary bile acids in both genotypes. There was also a large reduction in the proportion of TCA and TDCA in *Asbt^–/–^* mice following norUDCA treatment. The biliary bile acid hydrophobicity changes are reflected in the calculated hydrophobicity index, which decreased from +0.166 to –0.483 in *Asbt^–/–^* mice with norUDCA feeding but was largely unchanged in WT mice (calculated hydrophobicity index value of –0.453 versus –0.489 in chow- and norUDCA-fed mice, respectively). For comparison, the amounts of different bile acid species excreted into the feces are shown in Supplemental Figure 3. Under chow-fed conditions, the fecal bile acid content was approximately 5-fold greater in *Asbt^–/–^* versus WT mice and included a higher proportion of cholic acid and deoxycholic acid (DCA). Administration of norUDCA in the diet increased the fecal bile acid content in both WT and *Asbt^–/–^* mice and shifted the endogenous bile acid composition toward the 6-hydroxylated muricholate species. The increase in fecal bile acid levels was driven primarily by the exogenous norUDCA; however, the amount of endogenous bile acid in feces was also increased in both WT mice and *Asbt^–/–^* mice after administration of norUDCA.

The effect of norUDCA feeding on the output of other biliary solutes in WT and *Asbt^–/–^* mice are shown in Table 1. The total glutathione concentration and output tended to be higher in chow-fed *Asbt^–/–^* versus WT mice. This may be a mechanism to increase bile acid–independent bile flow to compensate for interruption of the bile acid enterohepatic circulation and a reduction in bile acid–dependent bile flow. Administration of norUDCA to WT and *Asbt^–/–^* mice did not change the biliary glutathione concentration but increased biliary glutathione output by 3- to 4-fold in both genotypes. Biliary cholesterol levels were slightly decreased in WT and *Asbt^–/–^* mice fed the norUDCA diet, but total cholesterol output was elevated versus chow-fed mice due to increases in bile flow. In contrast to biliary cholesterol, administration of norUDCA dramatically reduced biliary phospholipid secretion in both WT and *Asbt^–/–^* mice, in agreement with previous studies (1, 3, 4). This ineffective coupling of norUDCA and phospholipid secretion has been attributed to norUDCA’s lower surface activity and a reduced ability to extract phospholipid from the canalicular membrane (21). Overall, these findings argue that the ASBT is not required for the absorption of norUDCA or its ability to stimulate bicarbonate-rich hypercholeresis in mice.

**Figure 2:**
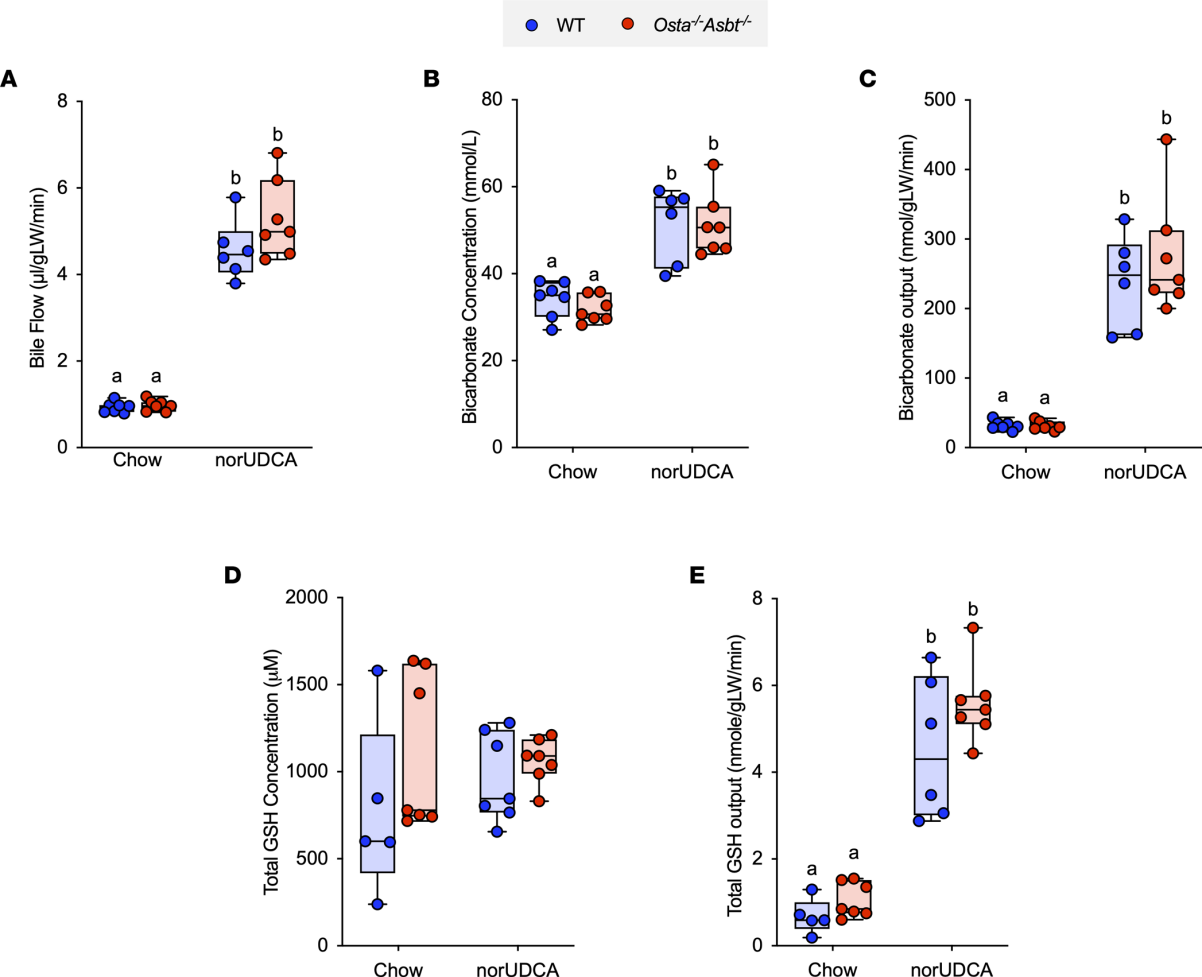
norUDCA treatment increases bile flow and biliary bicarbonate and solute output in WT and *Osta^–/–^ Asbt^–/–^* mice. (**A**) Bile flow. (**B**) Biliary bicarbonate concentration. (**C**) Bicarbonate output. (**D**) Glutathione concentration. (**E**) Glutathione output. Median values (line), interquartile range (boxes), and minimum to maximum values (whiskers) are shown; *n* = 5–7 mice per group. The data were evaluated for statistically significant differences using an ordinary 2-way ANOVA with a Tukey’s multiple-comparison test. Distinct lowercase letters indicate significant differences between groups (*P* < 0.05).

OSTα-OSTβ is a heteromeric bidirectional facilitative transporter and is responsible for bile acid and organic solute export across the basolateral membrane of various epithelium. Like the ASBT, OSTα-OSTβ is expressed by ileal enterocytes and cholangiocytes. However, OSTα-OSTβ is also expressed at lower levels in epithelium of the proximal small intestine and colon, where it may be involved in the export of bile acids that were taken up across the apical membrane by passive diffusion (28, 29). Due to their higher pKa versus taurine-conjugated bile acids, a fraction of unconjugated and glycine-conjugated bile acids are protonated in the lumen and can gain entry to the epithelium by nonionic diffusion (30). Once inside the cytoplasmic compartment, weak acids ionize at this neutral pH, potentially impeding their exit from the cell by passive diffusion and necessitating the requirement for an efflux carrier such as OSTα-OSTβ. To determine if OSTα-OSTβ may be contributing to the absorption and bicarbonate-rich choleresis induced by norUDCA, we examined bile flow and biliary bicarbonate output in background strain–matched WT and *Osta^–/–^ Asbt^–/–^* mice fed chow or chow plus 0.5% norUDCA for 7 days. *Osta^–/–^ Asbt^–/–^* mice were selected for these studies in place of *Osta^–/–^* mice because inactivation of the Asbt protects *Osta^–/–^* mice from ileal injury and attenuates the associated adaptive changes such as lengthening of the small intestine and ileal histological alterations such as villous blunting, increased numbers of mucin-producing cells, and increased cell proliferation (31). These phenotypic changes in *Osta^–/–^* mice were predicted to complicate interpretation of the findings with regard to the role of OSTα-OSTβ transport activity in the intestinal absorption and choleretic actions of norUDCA. The experimental scheme and morphological response to norUDCA administration is shown in Supplemental Figure 4. As with the WT and *Asbt^–/–^* mice, the liver weight/body weight ratio was increased in *Osta^–/–^ Asbt^–/–^* and matched WT mice with norUDCA treatment (Supplemental Figure 4E). Compared with chow-fed mice, administration of norUDCA increased bile flow rate by 5- to 6-fold, biliary bicarbonate concentration by 2-fold, bicarbonate output by more than 10-fold, and glutathione output by 5- to 6-fold in WT mice and background strain–matched mice lacking both ASBT and OSTα (Figure 2).

**Figure 3:**
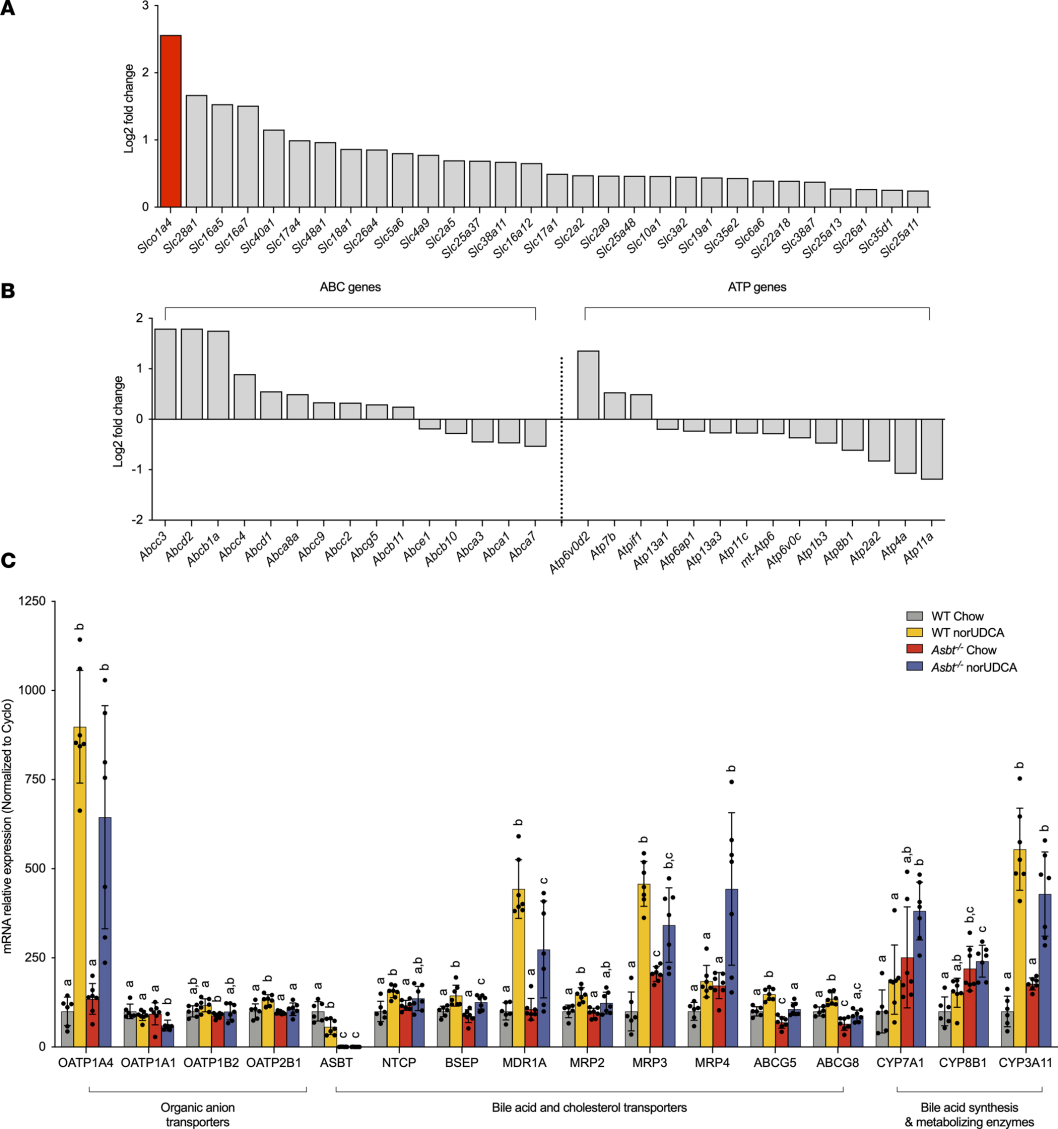
norUDCA treatment alters expression of a limited number of hepatic transporter genes. RNA-Seq analysis of livers from WT mice fed chow or the norUDCA-diet. (**A**) Differentially expressed *SLC* membrane transporter genes whose expression was significantly induced (*P* < 0.05; *n* = 6 per group) in norUDCA-treated versus chow mice. (**B**) Differentially expressed *ABC* transporter and *ATP* P-type ATPase genes (*P* < 0.05; *n* = 6 per group) in the norUDCA-treated versus chow mice. (**C**) Hepatic expression of the indicated transporters and bile acid–related biosynthesis or metabolizing enzymes in WT and *Asbt^–/–^* mice fed chow or the norUDCA-containing diet for 7 days. RNA was isolated from livers of individual mice and used for real-time PCR analysis. The mRNA expression was normalized using cyclophilin, and the results for each gene are expressed relative to chow-fed WT mice (set at 100%). Mean ± SD, *n* = 6–7 mice per group. The data were evaluated for statistically significant differences using an ordinary 1-way ANOVA with a Tukey’s multiple-comparison test. Distinct lowercase letters indicate significant differences between groups (*P* < 0.05).

The negative findings for ASBT and OSTα-ASBT–null mice do not exclude the potential involvement of other membrane transporters. We hypothesized that norUDCA may act in a feed-forward fashion to induce hepatocyte or cholangiocyte expression of transporters involved in norUDCA’s absorption, cholehepatic shunting, or mechanism of action. To identify potential candidates, RNA-Seq analysis was performed using livers from WT mice fed chow or norUDCA-containing diets. Using a log_2_(fold-change) > 1 and multiple testing (FDR 5%), 1,232 downregulated and 1,087 upregulated genes were identified in norUDCA-treated versus chow-fed WT mice. Narrowing our focus to the liver membrane transporter gene transcriptome revealed 80 solute carrier (*SLC*) family members, 15 ATP-binding cassette (*ABC*) family members, and 14 transporting P-type ATPases (*ATP*) family members (Figure 3, A and B, and Supplemental Figure 5A) that are differentially expressed in norUDCA versus chow-fed control WT mice. Of these hepatic genes, expression of 30 *SLC*, 10 *ABC*, and 3 *ATP-*type transporters was significantly induced. Among the most highly induced transporter genes in norUDCA-treated mice was *Slco1a4* (OATP1a4; originally called Oatp2). Members of the OATP1a/1b family, such as OATP1a4, are sodium-independent facilitative uptake carriers that mediate hepatocellular clearance of a variety of organic anions, steroids, sulfated, and glucuronidated metabolites (32). Notably, OATP1a4 has a substrate specificity that includes mainly unconjugated bile acids and is expressed on the sinusoidal membrane of perivenous hepatocytes (33).

To further pursue the RNA-Seq findings, real-time PCR was used to measure mRNA expression of select transporters and genes critical for bile acid homeostasis (Figure 3C). Administration of norUDCA induced OATP1a4 mRNA expression by more than 6-fold in WT and *Asbt^–/–^* mice, whereas other hepatic OATP family genes — OATP1a1, OATP1b2, and OATP2b1 — were largely unaffected. Regarding other transporters involved in bile acid or cholesterol metabolism, hepatic expression of Asbt was decreased, whereas NTCP (*Slc10a1*), BSEP (*Abcb11*), MRP2 (*Abcc2*), and *Abcg5/8* expression were modestly increased and MRP3 (*Abcc3*) RNA levels were increased by 3- to-4 fold (Figure 3C). In liver, expression of the bile acid biosynthetic genes *Cyp7a1* and *Cyp8b1* were significantly increased in *Asbt^–/–^* mice fed norUDCA versus WT mice (Figure 3C). In ileum, administration of norUDCA tended to reduce mRNA expression of the bile acid homeostasis–related genes for ASBT, FGF15, and OSTα-OSTβ but had little effect on IBABP (*Fabp6*) expression (Supplemental Figure 5B) in WT mice and did not affect expression of these genes in *Asbt^–/–^* mice. Notably, administration of norUDCA significantly induced expression of pregnane X-receptor (PXR) target genes, including *Slco1a4*, *Abcc3*, and *Cyp3a11*. These findings are in agreement with pathway analysis of the RNA-Seq data, which identified PXR-mediated direct regulation of xenobiotic metabolizing enzymes as one of the top-regulated pathways (Supplemental Figure 6A). To pursue this observation and directly test the hypothesis that norUDCA may be acting directly via PXR or other bile acid–activated nuclear receptors, the ability of norUDCA to activate mouse PXR, human farnesoid X-receptor (FXR), and human vitamin D receptor (VDR) was examined in transfected human Huh7 cells. Whereas the positive control compounds pregnenolone 16α-carbonitrile (PCN), GW4064, and 25-hydroxy vitamin D activated their cognate receptors in this assay, norUDCA did not increase the activity of PXR, FXR, or VDR reporter plasmids (Supplemental Figure 6B).

**Figure 4:**
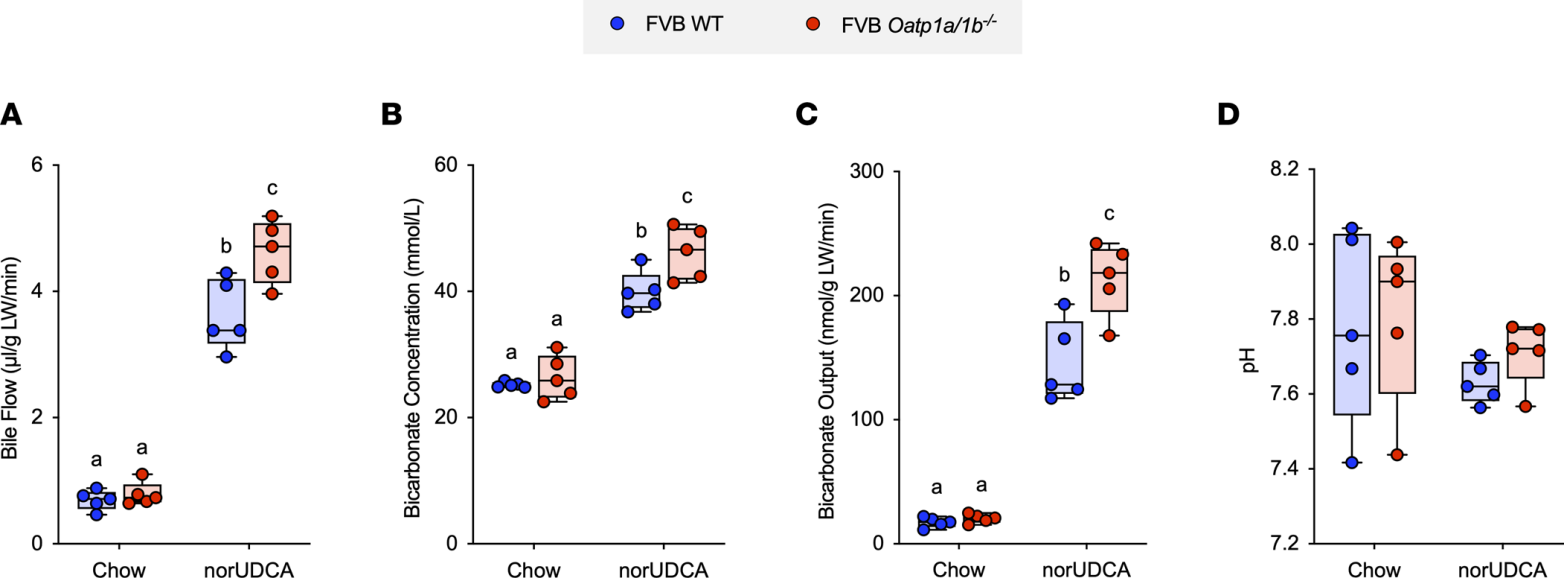
norUDCA treatment increases bile flow and biliary bicarbonate and solute output in WT and *Oatp1a/1b^–/–^* mice. (**A**) Bile flow. (**B**) Biliary bicarbonate concentration. (**C**) Bicarbonate output. (**D**) Biliary pH. Median values (line), interquartile range (boxes), and minimum to maximum values (whiskers) are shown; *n* = 5 mice per group. The data were evaluated for statistically significant differences using an ordinary 2-way ANOVA with a Tukey’s multiple-comparison test. Distinct lowercase letters indicate significant differences between groups (*P* < 0.05).

The significant increase observed for hepatic OATP1a4 expression raised the prospect that this transporter may be induced in a feed-forward fashion to facilitate hepatic clearance and cholehepatic shunting of norUDCA. To directly test that hypothesis, the ability of norUDCA to induce bile secretion and biliary bicarbonate output was examined in background strain–matched (FVB) WT and *Oatp1a/1b^–/–^* mice, in which *Slco1a1*, *Slco1a4*, *Slco1a5*, *Slco1a6*, and *Slco1b2* have been excised by cre-mediated deletion of the *Slco1a/1b* gene cluster (34). The well-characterized *Oatp1a/1b^–/–^* mouse model was selected for these studies since the OATP1a and OATP1b paralogs display considerable overlap in their tissue expression and substrate specificity, and these other members of the mouse OATP1a/1b subfamily could partially compensate for loss of OATP1a4 alone. The experimental scheme and morphological response to norUDCA feeding in *Oatp1a/1b^–/–^* mice are shown in Supplemental Figure 7. Administration of norUDCA to the FVB background WT and *Oatp1a/1b^–/–^* mice for 7 days decreased body weight for both genotypes and increased the liver weight and liver weight/body weight ratio in the WT but not *Oatp1a/1b^–/–^* mice. Bile flow and biliary solute output are shown in Figure 4. Bile flow and biliary bicarbonate concentration, bicarbonate output, and pH were similar in the chow-fed WT and *Oatp1a/1b^–/–^* mice. Like WT C57BL/6J mice, administration of norUDCA to WT FVB mice significantly increased bile flow by 4.7-fold, biliary bicarbonate concentration by 1.6-fold, and bicarbonate output by 7.5-fold as compared with chow-fed mice. In the *Oatp1a/1b^–/–^* mice, administration of norUDCA induced a 5-fold increase in bile flow rate, a 2-fold increase in biliary bicarbonate concentration, and an 11-fold increase in bicarbonate output, all of which were significantly higher than in the matched FVB WT mice.

**Figure 5:**
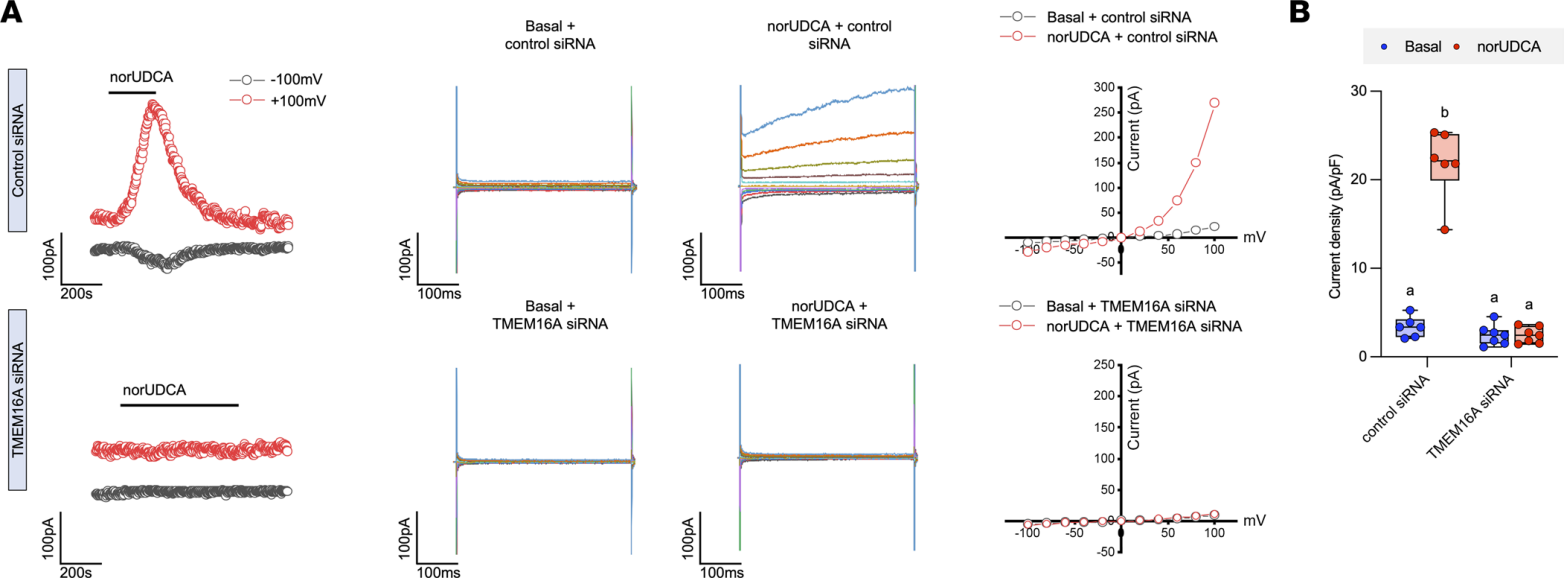
norUDCA stimulates Cl^–^ currents mediated by TMEM16A. (**A**) Representative whole-cell currents in MLC cells transfected with nontargeting siRNA or TMEM16A siRNA measured under basal conditions or during exposure to norUDCA (250 μM). Currents measured at –100 mV (black) or +100 mV (red), representing I_Cl_^–^ are shown. Compound exposure is indicated by the black bar. A voltage-step protocol from a holding potential of –40 mV, with 450 ms steps from –100 to +100 mV in 20 mV increments. Currents demonstrated time-dependent activation at membrane potentials +100 mV. The I-V plot was generated from these protocols during basal (black) and norUDCA-stimulated (red) conditions. (**B**) Cumulative data demonstrating maximum increase in current density (pA/pF) in response to norUDCA in the absence or presence of TMEM16A siRNA silencing; *n* = 5–6 cells per group. Median values (line), interquartile range (boxes), and minimum to maximum values (whiskers) are shown. The data were evaluated for statistically significant differences using an ordinary 2-way ANOVA with a Tukey’s multiple-comparison test.

**Figure 6:**
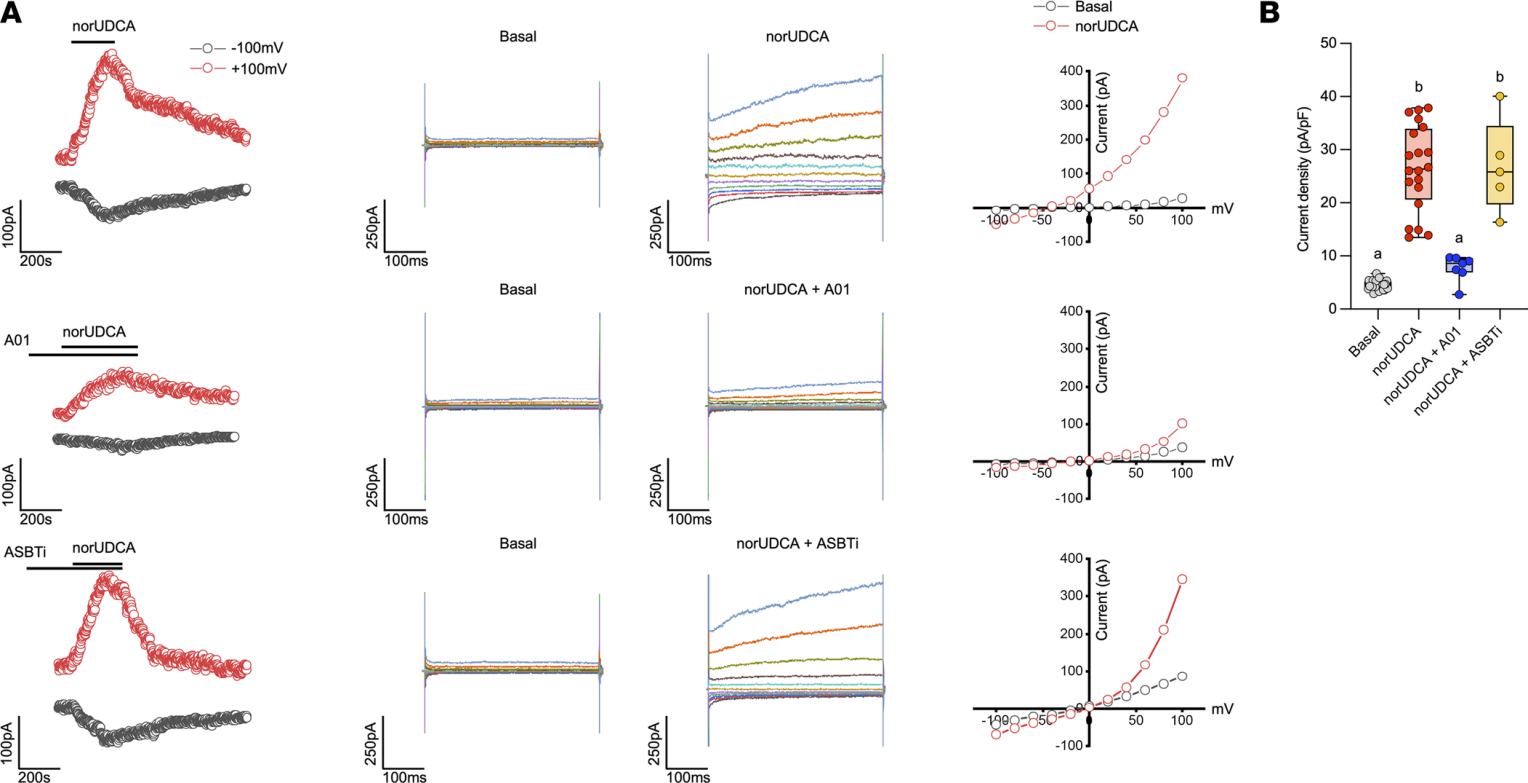
TMEM16A Cl^–^ current activation by norUDCA is independent of ASBT transport. (**A**) Representative whole-cell currents in MLC cells measured under basal conditions and during exposure to norUDCA (250 μM) following preincubation with vehicle (top), TMEM16A inhibitor (10 μM A01; middle), or ASBTi (100 nM SC-435; bottom). Currents measured at –100 mV (black) or +100 mV (red) representing I_Cl_^–^ are shown. Compound exposure is indicated by the black bar. The I-V plot was generated from these protocols during basal (black) and norUDCA-stimulated (red) conditions. (**B**) Cumulative data demonstrating maximum increase in current density (pA/pF) in response to norUDCA in the absence or presence of TMEM16A inhibitor or ASBTi; *n* = 5–35 cells per group. The data were evaluated for statistically significant differences using an ordinary 1-way ANOVA with a Tukey’s multiple-comparison test. Values with distinct superscript lowercase letters are significantly different (*P* < 0.05).

Our findings indicate that the major bile acid transporters ASBT, OSTα-OSTβ, and OATP1a/1b family members are not required for the choleretic activity of norUDCA. However, the question remains as to how norUDCA induces such a potent bicarbonate-rich hypercholeresis. The opening of cholangiocyte apical membrane Cl^–^ channels provides the critical driving force for biliary bicarbonate and fluid secretion (15). In addition to the cAMP-activated Cl^–^ channel CFTR (17), the Ca^2+^-activated Cl^–^ channel TMEM16A (ANO1) and the volume-regulated Cl^–^ channel leucine rich repeat-containing protein 8a (LRRC8A) have been shown to play important roles in cholangiocyte secretion (19, 35). We focused our studies on TMEM16A for the following reasons: (a) administration of norUDCA was still able to induce bile flow and biliary bicarbonate secretion In *Cftr^–/–^* mice (5), and (b) UDCA and TUDCA has been shown to stimulate cholangiocyte fluid secretion through the activation of TMEM16A Cl^–^ channels (19). Initial pilot experiments revealed that norUDCA potently stimulated Cl^–^ channel activity in mouse large cholangiocytes (MLCs) with the properties previously described for TMEM16A (19, 36). To confirm a role for TMEM16A, MLCs were cotransfected with a fluorescent oligonucleotide and a control siRNA or siRNA targeted to TMEM16A to reduce TMEM16A protein levels (Supplemental Figure 8) prior to patch clamping of the fluorescent oligonucleotide–labeled cells. As shown in Figure 5A and quantified in Figure 5B, knockdown of TMEM16A expression in MLCs blocked the ability of norUDCA to induce Cl^–^ currents. TMEM16A-dependent chloride secretion was further confirmed by preincubation with the TMEM16A inhibitor A01, which also abolished the norUDCA stimulation of Cl^–^ currents in these cells (Figure 6, A and B). Previous studies have shown that inhibition of the ASBT blocks stimulation of Cl^–^ currents in cholangiocytes by the conjugated bile acid TUDCA (19). In agreement with our in vivo studies showing that loss of the ASBT did not affect the bicarbonate-rich hypercholeresis induced by unconjugated norUDCA, preincubation of MLCs with the ASBT inhibitor SC-435 did not impact the norUDCA-stimulation of Cl^–^ channels (Figure 6, A and B).

Administration of norUDCA (3), ASBT inhibitors (37, 38), or a bile acid sequestrant (39) have shown benefit in the *Abcb4^–/–^* mouse model of cholestasis and appear to involve overlapping and complementary therapeutic mechanisms of action. Prompted by our findings for the norUDCA-fed *Asbt^–/–^* mice and SC-435–treated MLCs, we examined the effect of pharmacological interruption of the enterohepatic circulation of bile acids on the choleretic actions of norUDCA by coadministering an ASBT inhibitor (ASBTi) (SC-435) or bile acid sequestrant (colesevelam). Colesevelam is a second-generation bile acid sequestrant and nonabsorbable polymer that binds native bile acids through a combination of hydrophobic and ionic interactions and with a higher affinity than first-generation sequestrants such as cholestyramine (40). Male WT mice were fed chow, chow supplemented with 0.006% (w/w) ASBTi (SC-435), chow supplemented with 2% (w/w) colesevelam, or one of those diets plus 0.5% (w/w) norUDCA for 7 days. The experimental scheme and morphological response to norUDCA feeding is shown in Supplemental Figure 9. Administration of norUDCA to mice for 7 days tended to reduce body weight, particularly when coadministered with an ASBTi (Supplemental Figure 9, B and C), and tended to increase the liver weight/body weight ratio (Supplemental Figure 9E). However, the histology was assessed to be normal, and analysis of H&E-stained liver sections revealed no apparent histological differences between the treatment groups (Supplemental Figure 9F). The plasma chemistries were not significantly different between the groups (Supplemental Table 3). The levels of bile acids excreted into the feces for each of the treatment groups are shown in Supplemental Figure 9G. Administration of the ASBTi versus colesevelam resulted in a greater increase in the fecal bile acid content; however, both treatments induced similar changes in fecal bile acid composition versus chow control mice (Supplemental Figure 9H). Administration of norUDCA in the diet increased the fecal bile acid content in all treatment groups, due to increased excretion of the exogenous norUDCA and endogenous bile acids.

**Figure 7:**
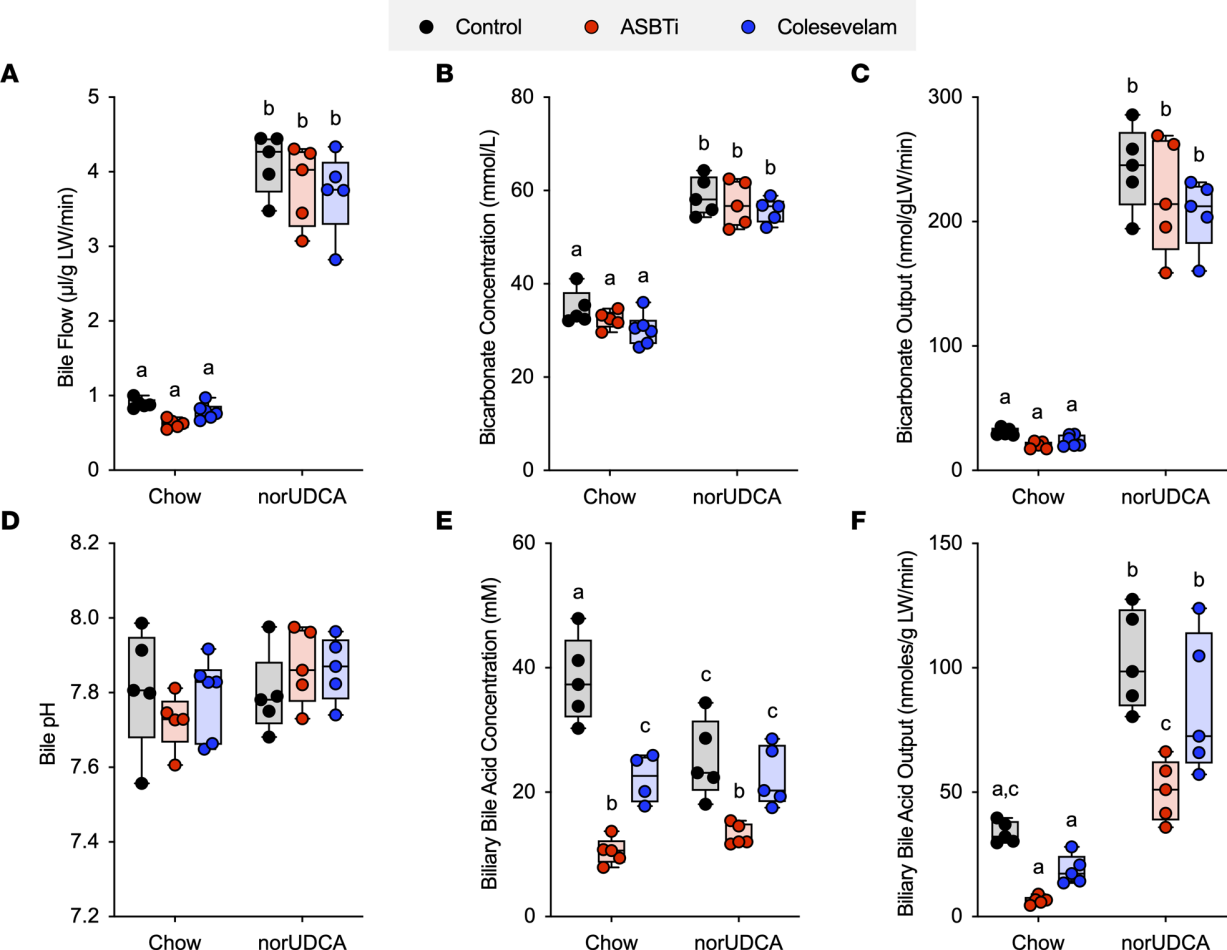
Pharmacological inhibition of intestinal bile acid absorption does not alter norUDCA induction of a bicarbonate-rich hypercholeresis in mice. (**A**) Bile flow. (**B**) Biliary bicarbonate concentration. (**C**) Bicarbonate output. (**D**) Biliary pH. (**E**) Biliary bile acid concentration. (**F**) Biliary bile acid output. Median values (line), interquartile range (boxes), and minimum to maximum values (whiskers) are shown; *n* = 5 mice per group. The data were evaluated for statistically significant differences using an ordinary 2-way ANOVA with a Tukey’s multiple-comparison test. Distinct lowercase letters indicate significant differences between groups (*P* < 0.05).

Like the findings for *Asbt^–/–^* mice, norUDCA significantly increased bile flow by 3- to 4-fold, bicarbonate concentration by about 2-fold, and bicarbonate output by about 8-fold in ASBTi-treated WT mice (Figure 7, A–D). Remarkably, norUDCA also stimulated a similar bicarbonate-rich choleresis when coadministered with colesevelam. In agreement with the block in intestinal absorption of bile acids, biliary bile acid concentrations were reduced in ASBTi- and colesevelam-treated versus control chow mice; however, administration of norUDCA significantly increased biliary bile acid output in all the treatment groups (Figure 7, E–F). The observation that coadministration of colesevelam did not attenuate the norUDCA-induced hypercholeresis prompted us to examine of the ability of colesevelam to bind norUDCA versus endogenous bile acids in simulated small intestinal fluid. In accord with previous findings, colesevelam efficiently bound glycodeoxycholic acid (GCDCA) and TDCA in vitro (41). However, under the same conditions, there was minimal binding of norUDCA to colesevelam (Supplemental Figure 10), providing a potential explanation for the inefficacy of coadministered colesevelam to antagonize the actions of norUDCA. In summary, pharmacological inhibition of intestinal bile acid absorption does not impede norUDCA’s ability to induce a bicarbonate-rich hypercholeresis in mice.

## Discussion

When first characterized by Hofmann and colleagues, side chain–shortened dihydroxy bile acids such as norCDCA, norDCA, and norUDCA were shown to induce a bicarbonate-rich bile flow that could not be accounted for by existing theories for bile formation. This led the authors to propose a model involving cholehepatic shunting of unconjugated nor-bile acids (1, 13, 22). Although norUDCA is currently in therapeutic development for the treatment of liver disease (8, 9, 42), important questions remain regarding the molecular mechanisms underlying the biliary bicarbonate secretion induced by norUDCA and its hypothesized cholehepatic shunting. The major findings of this study are (a) the major bile acid carriers ASBT, OSTα-OSTβ, and OATP transporters are not required for orally administered norUDCA to stimulate a bicarbonate-rich hypercholeresis, and (b) norUDCA stimulates the Ca^2+^-activated Cl^–^ channel TMEM16A in mouse cholangiocytes.

In our studies, norUDCA did not require ASBT activity to activate TMEM16A chloride channels, which are thought to be the final common pathway responsible for bile acid–stimulated biliary ductal secretion. In this paradigm, norUDCA is proposed to induce extracellular release of ATP, which activates surface P2 purinergic receptors and signals through the IP3 receptor to increase cytosolic Ca^2+^ and stimulate TMEM16A Cl^–^ secretion (15, 19, 43). This increases bicarbonate and fluid secretion in the biliary epithelium by driving Cl^–^/HCO_3_^–^ exchange via the anion exchange protein AE2 (*SLC4A2*) (18, 20, 44). Interestingly, the UDCA stimulation of cholangiocyte ATP release is thought to require CFTR and may act by stimulating the trafficking and fusion of ATP-containing vesicles with the apical membrane (45–47). However, norUDCA-induced stimulation of biliary bile flow and bicarbonate secretion is largely CFTR independent (5). This raises the prospect that norUDCA may be stimulating nucleotide release or bicarbonate secretion by additional mechanisms that will need to be explored in future studies.

The physiologic properties and metabolism of side chain–shortened C-23 bile acids such as norUDCA have been the subject of study (1, 21, 22). Due to a higher critical micelle concentration (CMC) (17 mM) than many natural bile acids, norUDCA is more likely present in monomeric rather than micellar form in bile (48). Based on their findings, Hofmann and coworkers proposed that C-23 nor-dihydroxy bile acids are sufficiently hydrophobic to be absorbed by passive diffusion (14). However, membrane transporters participate in the intestinal absorption and hepatic clearance of various hydrophobic drugs and endobiotics, including unconjugated C-24 bile acids (34, 49). To date, study of the contribution of individual bile acid and organic anion carriers to the transport of norUDCA has been largely restricted to transfected cell–based models (27). The ASBT and OSTα-OSTβ play central roles in the intestinal reabsorption of bile acids and are also expressed by the biliary epithelium (31–33). As such, it was possible that these major bile acid transporters could play a direct or indirect role in the absorption, cholehepatic shunting, and choleretic actions of norUDCA.

In agreement with previous studies, administration of norUDCA significantly increased bile flow and bicarbonate concentration in WT mice, and similar results were observed for *Asbt^–/–^* and *Osta^–/–^ Asbt^–/–^* mice. Biliary bile acid output, typically lower in *Asbt^–/–^* versus WT mice, was significantly increased by administration of norUDCA. This was driven primarily by the increase in bile flow, since the biliary total bile acid concentration was not significantly changed. However, norUDCA administration significantly altered the biliary bile acid composition, with norUDCA accounting for more than half the biliary bile acid species and a concomitant reduction in endogenous bile acid species. In *Asbt^–/–^* mice and WT mice treated with ASBT inhibitors, the biliary bile acid composition becomes more hydrophobic and enriched in TCA and TDCA. This is most likely due to increased hepatic TCA synthesis and an increased flux into the colon, where TCA is metabolized to DCA, passively reabsorbed, and carried back to the liver for uptake and reconjugation in hepatocytes (38, 50, 51). Following administration of norUDCA, the biliary endogenous bile acid composition in WT and *Asbt^–/–^* mice become remarkably similar. Interestingly, there was an increase in fecal endogenous bile acids observed in WT mice treated with norUDCA, and it may be secondary to decreased ileal ASBT expression or weak inhibition of ileal ASBT transport activity by norUDCA. Using cell-based models, hydrophobic unconjugated bile acids, such as CDCA, DCA, UDCA, LCA, exhibited little apparent ASBT-mediated uptake over background but are still able to compete for conjugated bile acid uptake (52).

We hypothesized that norUDCA may induce hepatic expression of its own transporters in a feed-forward fashion to facilitate cholehepatic shunting. RNA-Seq analysis revealed that norUDCA induced expression of only a small subset of hepatic transporter genes, including several transporters involved in bile acid homeostasis — OATP1a4, MRP3, MRP4, and MDR1a. As previously observed (3, 6), many of the hepatic genes induced by norUDCA are Phase1/2 enzymes and PXR targets, raising the prospect that norUDCA may be acting directly via PXR. However, when directly investigated, norUDCA did not activate mouse PXR or the bile acid sensing nuclear receptors, FXR, or VDR as measured using nuclear receptor-luciferase reporter assays in transfected human hepatoma Huh7 cells. These findings are in agreement with recent results showing that norUDCA did not activate or inhibit FXR in HepG2 cells transfected with a FXR reporter plasmid (53) and are consistent with previous data (6). Other hepatic gene expression pathways that were significantly induced by administration of norUDCA included those regulated by the nuclear receptor constitutive androstane receptor (CAR; *NR1L3*). Unlike PXR, FXR, or VDR, bile acids do not directly bind and activate CAR (54) but may act indirectly through a ligand-independent mechanism to stimulate CAR nuclear translocation (55). However, the role of CAR and other xenobiotic sensors in the actions of norUDCA remain to be investigated.

We focused our attention on OATP1a4, since its expression was most highly induced in the hepatic transporter transcriptome of norUDCA-fed WT mice. OATP1a4 (originally called OATP2) is expressed on the hepatocyte sinusoidal membrane, transports a variety of organic anions, and contributes to the hepatic clearance of steroid sulfates, bile acids, and drugs (56–58). However, the 3 most abundant hepatic OATP isoforms in rodents — OATP1a1, OATP1b2, and OATP1a4 — exhibit overlapping substrate specificity (32), prompting us to use the *Oatp1a/1b^–/–^* mouse model lacking all OATP1a/1b transporters for our studies (34). Like *Asbt^–/–^* and *Osta^–/–^ Asbt^–/–^* mice, loss of the OATP1a/1b transporters did not impair the ability of norUDCA to stimulate a bicarbonate-rich hypercholeresis. This is in line with studies using cells stably expressing human liver OATPs that failed to detect appreciable norUDCA transport by human OATP1B1, OATP1B3, or OATP2B1 (27). The finding that biliary bicarbonate concentration and bile flow increases in the norUDCA-fed WT and various transporter KO models is strongly consistent with the mechanism for cholehepatic shunting of norUDCA proposed by Hofmann and colleagues (1), but our study also had several limitations. This included the use of a high pharmacological dose of norUDCA administered in the diet. Although widely used for previous studies in mice (3–5), the higher dose may diminish the contribution of saturable carrier-mediated mechanisms to the absorption and actions of norUDCA. Another limitation of the study is that only the unmodified norUDCA was quantified and that metabolites such as norUDCA glucuronides were not measured (3, 21). However, together with previous reports (27, 59), our studies support the concept that much of the enterohepatic cycling and cholehepatic shunting of norUDCA is passive and does not require the ASBT, OSTα-OSTβ, NTCP, OATPs, or MRP2. It is still possible that canalicular transporters such as the bile salt export pump (*ABCB11*), MDR1 (*ABCB1*), or other ABC transporters are involved in the hepatocyte secretion of unmodified norUDCA into bile. In that regard, the mRNA expression of MDR1 and BSEP are increased in norUDCA-treated mice.

One of the most translational findings in this study is the ability of norUDCA to increase bile flow and biliary bicarbonate when coadministered with an ASBTi or bile acid sequestrant. Based on our findings with the *Asbt^–/–^* mice, it was not surprising that the choleretic actions of norUDCA were unaffected by an ASBTi. However, coadministration of norUDCA with a bile acid sequestrant had not been previously examined. This contrasts with UDCA, whose interactions with bile acid sequestrants have been studied in vitro, in animal models, and in human subjects (60–62). In those studies, cholestyramine and colestimide efficiently bound and reduced the intestinal absorption of coadministered UDCA. Therefore, it was surprising that coadministration of colesevelam did not attenuate the choleretic actions of norUDCA. Using in vitro assays, colesevelam bound norUDCA poorly versus conjugated bile acids; however, additional studies will be required to determine if this is a general property of all bile acid sequestrants. Although both ASBT inhibition and administration of bile acid sequestrants interrupt the enterohepatic circulation of bile acids, there are differences between the mechanisms of action by which they improve features of cholestasis (37–39, 63). In the present study, ASBT inhibition reduces total biliary bile acid concentrations, yet in the presence of norUDCA, bile flow was still induced. These findings indicate that pharmacological ileal ASBT inhibition does not antagonize norUDCA’s positive effects on bile flow and that, in certain settings, the 2 therapeutic approaches may have beneficial synergistic effects in cholestatic models (64). This includes a reduced biliary bile acid concentration, a more hydrophilic biliary bile acid composition, and an elevated biliary bicarbonate concentration observed with norUDCA plus ASBT inhibition versus ASBT inhibition alone.

Collectively, our findings demonstrate that norUDCA does not require the major bile acid transporters, ASBT and OSTα-OSTβ, or members of the OATP1a/1b family to induce a bicarbonate-rich hypercholeresis and can activate TMEM16A in an ASBT-independent fashion. However, even with norUDCA’s ability to stimulate TMEM16A, the magnitude of the increase suggests that multiple rounds of cholehepatic shunting are required to present the cholangiocyte with sufficient norUDCA to drive the bicarbonate and fluid secretion observed in vivo. The superior hypercholeretic activity of norUDCA is likely dependent upon its ability to evade hepatic amidation with taurine or glycine, whereas the therapeutic bile acid UDCA is efficiently conjugated and competes with other native bile acids for carrier-mediated uptake. Finally, these results also provide support for further investigation of the therapeutic potential of a combination of norUDCA and blockers of the enterohepatic circulation of bile acids in cholestatic liver disease.

## Methods

### Materials.

norUDCA was received as a research gift from Falk Pharma to Michael Trauner. SC-435 was received as a research gift from Shire Pharmaceuticals. Colesevelam was provided by Alan Hofmann (UCSD, San Diego, California, USA). TMEM16A inhibitor (TMEM16Ainh-A01) was purchased from MilliporeSigma. Huh7 cells were obtained from ATCC. The MLC cell line had been provided by Gianfranco Alpini and Shannon Glaser (Baylor Scott & White Disease Research Center, Baylor Scott & White Healthcare, Temple, Texas, USA).

### Animals.

The *Asbt^–/–^* mice (*C57BL/6NJ-Slc10a2^tm1a(KOMP)Mbp^*; Asbt KO-first, reporter-tagged insertion with conditional potential; Targeting Project CSD76540; https://www.komp.org/ProductSheet.php?cloneID=617849) were obtained from the Knockout Mouse Project (KOMP) Baylor College of Medicine Repository (Houston, Texas USA), and colonies of *Asbt^–/–^* and matched WT mice were maintained at the Emory University School of Medicine. Characterization of the ileal and liver Asbt mRNA expression, fecal bile acid excretion, and bile acid pool size and composition in male and female WT and Asbt KO-first mice is shown in Supplemental Figure 1. The matched background strain WT and *Osta^–/–^ Asbt^–/–^* mice were generated from crossbreeding *Osta^–/–^* and *Asbt^–/–^* mice that had been backcrossed for 8 generations onto a C57BL/6J background, as described previously (31). Male OATP1a/1b gene cluster KO mice (*Oatp1a/1b^–/–^*) (FVB.129P2-Del[Slco1b2-Slco1a5]1Ahs) (34) and background-matched WT FVB mice were purchased from Taconic Biosciences. For the ASBTi and colesevelam studies, WT male mice (C57BL/6J; 000664) mice were obtained from The Jackson Laboratory.

### Animal treatments and bile flow measurements.

All experiments were performed using male mice, 3 months of age (25–30 g body weight). The indicated genotypes were fed rodent chow (Envigo; Teklad custom diet No. TD.160819; global 18% protein rodent diet) for 7 days. For the next 7 days, the mice were fed TD.160819 rodent chow or TD.160819 rodent chow containing the indicated combinations of 0.5% (w/w) norUDCA, 0.006% (w/w) ASBTi (SC-435; dose ~11 mg/kg/d), or 2% (w/w) colesevelam. The amount of norUDCA, ASBTi, and colesevelam administered was selected based on published studies demonstrating sufficient doses to induce bile flow (norUDCA) or disrupt the enterohepatic circulation of bile acids (ASBTi, colesevelam) (5, 39, 65). Based on an estimate of 3 g of diet consumed per day per 25 g body weight, the dose of norUDCA was approximately 600 mg/kg/day. Bile flow was measured in mice as previously described (3). At the end of the bile collection period, blood was obtained by cardiac puncture to measure plasma chemistries. Portions of the liver were taken for histology and measurements of gene expression.

### Plasma biochemistries and biliary solute measurements.

Plasma chemistries were measured at the Emory University Department of Animal Resources Quality Assurance and Diagnostic Laboratory. The bile samples were used immediately after isolation to measure HCO_3_^–^, pH, total CO_2_, Na^+^, K^+^, Cl^–^, and glucose using a blood gas analyzer (i-STAT; Abbott Point of Care Inc.) in the Clinical Pathology Laboratory, Emory University-Yerkes National Primate Research Center. Biliary glutathione concentrations were measured in the Emory University Department of Pediatrics Biomarkers Core Facility. Biliary bile acid, cholesterol, and phospholipid concentrations were measured enzymatically as previously described (51, 66).

### Histological analysis.

The liver segments were fixed in 10% neutral formalin (Sigma-Aldrich), embedded in paraffin, and processed by Children’s Healthcare of Atlanta Pathology Services. Histological sections (5 μm) were cut and stained with H&E. The liver histology was assessed in a blinded fashion by a certified veterinary pathologist.

### Bile acid measurements.

To characterize the *Asbt^–/–^* mice, feces were collected from single-housed adult male and female mice over a 72-hour period. The total fecal bile acid content was measured by enzymatic assay (51, 67). Pool size was determined as the bile acid content of the small intestine, liver, and gallbladder removed from nonfasted mice (68, 69). Quantitative analysis of the biliary bile acids from chow or norUDCA-fed mice was carried out at the Clinical Mass Spectrometry Laboratory at Cincinnati Children’s Hospital Medical Center as described (70). For the norUDCA feeding studies, fecal samples were collected from cages of group-housed mice with standard bedding at the end of the 7-day chow or norUDCA feeding period. Fecal bile acid composition was determined using a Hewlett-Packard Agilent gas chromatography/mass spectrometer in the Department of Pediatrics Biomarkers Core Facility at Emory University as described (71).

### RNA-Seq analysis.

Total RNA was extracted from frozen liver tissue using TRIzol reagent (Invitrogen). RNA-Seq libraries were prepared by Novogene Co. and sequenced on an Illumina HiSeq1000 system. Differential expression analysis was performed using the DESeq2 R package of Bioconductor (72). The resulting *P* values were adjusted using the Benjamini-Hochberg procedure to control for the FDR (73). Differentially expressed genes with a fold change > 1.0 and adjusted *P* < 0.05 were selected for functional annotation (Gene Expression Omnibus [GEO], GSE145020). Pathway analysis of the RNA-Seq data was performed using MetaCore (GeneGo Inc.).

### Luciferase assays.

The human hepatocellular carcinoma–derived cell line Huh7 were a gift from the MK Estes laboratory (Department of Molecular Virology and Microbiology, Baylor College of Medicine; Houston, Texas, USA) (74). The Huh7 cells were transfected with expression plasmids for a chimeric nuclear receptor encoding the ligand binding domain of mouse PXR fused to the DNA binding domain of GAL4 along with a 5***×*** Upstream Activation Sequence–luciferase (UAS-luciferase) reporter, or expression plasmids for human FXR or VDR, along with FXR- or VDR-responsive luciferase reporter plasmids. Ligand additions and measurements of luciferase activity were performed as described (65).

### TMEM16A silencing.

TMEM16A siRNA (TMEM16A-HSS123904) was used to inhibit TMEM16A expression in whole-cell patch-clamp experiments as previously described (19). The control or TMEM16A siRNA was cotransfected with a Block-it TM Fluorescent Oligo (catalog 2013, Invitrogen) to identify the oligonucleotide transfected cells for whole-cell patch clamp current recording. The degree of TMEM16A silencing was evaluated by Western blot analysis using anti–mouse TMEM16A antibody (1:1000, Alomone Laboratories, ACL-011) (19).

### Measurement of Cl^–^ currents.

Studies were performed in MLC, which had been isolated from normal mice (BALB/c) and immortalized by transfection with the SV40 large-T antigen gene as previously described (75). Membrane currents were measured by whole-cell patch-clamp techniques with a standard extracellular and intracellular (pipette) solution. Recordings were made with an Axopatch 200B amplifier (Axon Instruments) and digitized and analyzed using pCLAMP version 11.0.3 as described (45). Two voltage protocols were utilized: (a) holding potential of –40 mV, with ramp protocol from –100mV to +100mV for duration 450 ms at 2-second intervals; (b) holding potential –40 mV, with 450 ms steps from –100 mV to +100 mV in 20 mV increments. Protocol 1 was utilized for real-time tracings, and protocol 2 was used for generation of current-voltage (I-V) plots. Results are reported as current density (pA/pF) to normalize for differences in cell size. Details of the buffer solutions, voltage protocols, and data acquisition are described in Supplemental Methods.

In vitro colesevelam bile acid binding assay. Bile acid binding to colesevelam was carried out as described (41).

### Data availability.

The liver RNA-Seq data set is available from the GEO repository with accession no. GSE145020.

### Statistics.

For the box and whisker plots, median values (line), interquartile range (boxes), and minimum to maximum values (whiskers) are shown. For the liver BA composition analysis and gene expression, mean ± SD is shown. The data were evaluated for statistically significant differences using the Mann-Whitney *U* test, the 2-tailed Student’s *t* test, 2-way ANOVA (with a Tukey-Kramer honestly significant difference post hoc test) or Sidak’s multiple-comparison test (GraphPad Prism). Values with different superscript letters are significantly different (*P* < 0.05).

### Study approval.

All animal experiments were approved by the Emory University IACUC in accordance with NIH guidelines for the ethical treatment of animals.

## Author contributions

JL, JKT, SJK, CDF, APF, MT, and PAD designed the study and conceived the experiments. JL, JKT, QL, KP, AR, SG, APF, and PAD performed experiments, collected results, and analyzed the data. JL, JKT, and PAD managed the study. JKT, JL, APF, MT, SJK, and PAD wrote the paper. JL and JKT contributed equally to this work. JL initiated the project before JKT joined the laboratory. After joining the lab, JKT worked with JL to perform experiments, collect results, and analyze data. JKT continued the work and completed the project after JL left the laboratory to accept another position. All authors read and approved the final manuscript.

## Supplementary Material

Supplemental data

## Figures and Tables

**Table 1:**
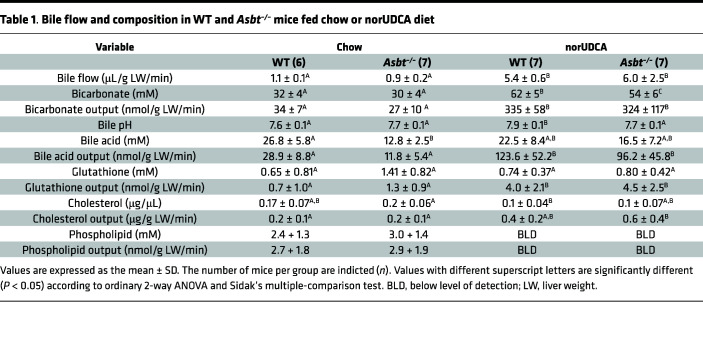
Bile flow and composition in WT and *Asbt^–/–^* mice fed chow or norUDCA diet

## Version 1 02/14/2023

In-Press Preview

## Version 2 03/22/2023

Electronic publication
