# Supplementary material for: Active enterohepatic cycling is not required for the choleretic actions of 24-norUrsodeoxycholic acid in mice
Source: JCI Insight. 2023 Mar 22;8(6):e149360. doi: 10.1172/jci.insight.149360 (PMC10070106; doi:10.1172/jci.insight.149360)
Supplement: Supplemental data [file jciinsight-8-149360-s108.pdf]

Supplemental Material

Abbreviations: ABC, ATP-binding cassette; ALP, alkaline phosphatase; ALT, alanine aminotransferase; ANO1, anoctamin-1; ASBT, apical sodium-dependent bile acid transporter; ASBTi, ASBT inhibitor; AST, aspartate aminotransferase; ATP, P-type ATPase; BSEP, bile salt export pump; CA, cholic acid; CAR, constitutive androstane receptor; CFTR, cystic fibrosis transmembrane conductance regulator; CHOL, cholesterol; DCA, deoxycholic acid; FGF15, fibroblast growth factor 15; FXR, farnesoid X-receptor; IBABP, ileal bile acid binding protein; LCA, lithocholic acid; LRRC8A, leucine-rich repeat-containing protein 8a; MDR2, multidrug resistance protein 2; MLC, mouse large cholangiocyte; OST, organic solute transporter; Ntcp, Na-taurocholate co-transporting polypeptide; OATP, organic anion transporting polypeptide; PCN, pregnenolone 16α-carbonitrile; PSC, Primary Sclerosing Cholangitis; PXR, pregnane X-receptor; siRNA, small interfering RNA; TBILI, total bilirubin; TCA, taurocholic acid; TCDCA, taurochenodeoxycholic acid; TDCA, taurodeoxycholic acid; TMEM16A, transmembrane member 16A; THDCA, taurohyodeoxycholic acid; TαMCA, tauro-α-muricholic acid; TβMCA, tauro-β-muricholic acid; TωMCA, tauro-ω-muricholic acid; TUDCA, tauroursodeoxycholic acid; TP, total protein; VDR, vitamin D receptor; WT, wild type.

Expanded Methods

Materials

norUDCA (24-*nor*-5β-cholan-23-oic acid) was received as a research gift from Dr. Falk Pharma to Dr. Michael Trauner. The purity of the norUDCA was confirmed by HPLC analysis. The ASBT inhibitor SC-435; (4R,5R)-5-[4-[4-(1-aza-4-azoniabicyclo[2.2.2]octan-4-yl)butoxy] phenyl]-3,3-dibutyl-7,8-dimethoxy-1,1-dioxo-4,5-dihydro-2H-1λ6-benzothiepin-4-ol) was received as a research gift from Shire Pharmaceuticals, and the ASBT inhibitory activity was confirmed using MDCK cells stably transfected with the human ASBT (IC_{50} ~18 nM). Colesevelam was provided

by Dr. Alan Hofmann (University of California at San Diego) and was received as a research gift from GelTex Pharmaceuticals/Genzyme. TMEM16A inhibitor (TMEM16Ainh-A01) 2-(5-ethyl-4-hydroxy-6-methylpyrimidin-2-ylthio)-N-(4-(4-methoxyphenyl) thiazol-2-yl) acetamide was purchased from Millipore Sigma (Cat #613551). Human hepatocellular carcinoma-derived cell line Huh7 were provided by Dr. Mary K. Estes (Department of Molecular Virology and Microbiology, Baylor College of Medicine; Houston, TX) (1). The mouse large cholangiocyte (MLC) cell line had been provided by Drs. Gianfranco Alpini and Shannon Glaser (Baylor Scott & White Disease Research Center, Baylor Scott & White Healthcare, Temple, TX).

Animals and Tissue Collection

All animal experiments were approved by the Institutional Animal Care and Use Committees at Emory University. The *Asbt*$^{-/-}$ mice (*C57BL/6NJ-Slc10a2*$^{tm1a(KOMP)Mbp}$; ASBT knockout-first, reporter-tagged insertion with conditional potential; Targeting Project CSD76540; https://www.komp.org/ProductSheet.php?cloneID=617849) were obtained from the Knockout Mouse Project (KOMP) Baylor College of Medicine Repository, and colonies of *Asbt*$^{-/-}$ and matched wild type (WT) mice generated from *Asbt*$^{+/-}$ crosses are maintained at the Emory University School of Medicine. The ASBT knockout-first mice were selected for these studies in place of the *Slc10a2*$^{tm1pda/J}$ whole body knockout mice (2) because they are in a pure C57BL/6NJ background and can be used to generate ASBT-floxed and tissue-specific null mice. Characterization of the ileal and liver ASBT mRNA expression, fecal bile acid excretion, and bile acid pool size in male and female WT and ASBT knockout-first mice is shown in Supporting Figure 1. The *Osta*$^{-/-}$*Asbt*$^{-/-}$ and background-matched WT mice were generated from crossbreeding of *Osta*$^{-/-}$ and *Asbt*$^{-/-}$ mice that had been backcrossed for 8 generations onto a C57BL/6J background, as described previously (3). *Osta*$^{-/-}$*Asbt*$^{-/-}$ mice were selected for these studies in place of *Osta*$^{-/-}$ mice because inactivation of the ASBT protects *Osta*$^{-/-}$ mice from ileal injury and

prevents/attenuates the associated adaptive changes including lengthening of the small intestine, ileal hypertrophy, villous blunting, increased numbers of mucin-producing cells, and increased cell proliferation (3). These adaptive changes were predicted to complicate interpretation of the findings with regards to the role of OSTα-OSTβ transport activity in the intestinal absorption and the choleretic actions of norUDCA. Male OATP1a/1b gene cluster knockout mice (*Oatp1a/1b*$^{-/-}$) (FVB.129P2-Del(Slco1b2-Slco1a5)1Ahs) and background-matched WT mice were purchased from Taconic Biosciences. For the ASBTi and colesevelam studies, WT male mice (C57BL/6J; #000664) were obtained from Jackson Labs. The mice were group-housed in ventilated cages (Super Mouse 750 Microisolator System; Lab Products) containing bedding (1/8" Bed-O-Cobbs; Andersons Lab Bedding Products) at temperature (22ºC) in the same 12h/12h light/dark cycle-controlled room of animal facility to minimize environmental differences and fed ad libitum rodent chow (13% of calories as fat; PicoLab Rodent Diet 20; LabDiet).

Animal Treatment and Bile Flow Measurements

All norUDCA experiments were performed using male mice, 3 months of age (approximately 25-30 g body weight). The indicated genotypes were fed rodent chow (Envigo; Teklad custom Diet No. TD.160819; global 18% protein rodent diet) for 7 days. For the next 7 days, the mice were fed TD.160819 rodent chow or TD.160819 rodent chow containing the indicated combinations of 0.5% (w/w) norUDCA, 0.006% (w/w) ASBTi (SC-435; dose ~11 mg/kg/day), or 2% (w/w) colesevelam. The amount of norUDCA, ASBTi, and colesevelam administered was selected based on published studies demonstrating these doses are sufficient to induce bile flow (norUDCA) or disrupt the enterohepatic circulation of bile acids (ASBTi, colesevelam) (4-6). Based on an estimate of 3 g of diet consumed per day per 25 g body weight, the dose of norUDCA administered is approximately 15 mg per 25 g mouse per day (600 mg/kg/day). At the end of the 7-day treatment period, bile flow in the mice was measured as previously described (7). Briefly, non-fasted mice were anesthetized using an isoflurane/O_2 mixture, placed on a 37°C heating pad

and maintained under isoflurane anesthesia during the procedure. Following laparotomy, the common bile duct was ligated, and gallbladder was cannulated. Residual gallbladder bile and hepatic bile secreted during the first 10 minutes of the collection period was discarded. Bile was then collected under a layer of mineral oil in pre-weighed tubes on ice for 120 minutes. Bile flow was determined gravimetrically and normalized to liver weight.

At the end of the bile collection period, blood was obtained by cardiac puncture to measure plasma chemistries. Liver wet weight was recorded at the time of necropsy. Portions of the liver were flash-frozen in liquid nitrogen and stored at -80º C for analysis of gene expression or fixed in 10% neutral formalin (Sigma-Aldrich) for embedding in paraffin and histological analysis. Small intestine was divided into 5 equal length segments and flushed with ice-cold phosphate-buffered saline (PBS) to remove luminal contents before weighing and flash-freezing in liquid nitrogen.

Plasma Biochemistries and Biliary Solute Measurements

Plasma chemistries, including total protein, albumin, alkaline phosphatase, alanine aminotransferase, aspartate aminotransferase, gamma-glutamyl transpeptidase, total bilirubin and cholesterol were measured at the Emory University Department of Animal Resources Quality Assurance and Diagnostic Laboratory. Immediately after isolation, the bile samples were used for determination of HCO_3^-, pH, total CO_2, Na^+, K^+, Cl^-, and glucose using a blood gas analyzer (i-STAT; Abbott Point of Care Inc) in the Clinical Pathology Laboratory, Emory University-Yerkes National Primate Research Center. Biliary glutathione concentrations were measured by HPLC in the Emory University Pediatrics Biomarkers Core Facility as described previously (8). Biliary bile acid and cholesterol concentrations were measured enzymatically as previously described (2, 9). In order to quantify biliary phospholipids, bile samples underwent chloroform-methanol extraction, followed by digestion with perchloric acid, and enzymatic assay to measure inorganic phosphate at the Wake Forest School of Medicine Lipid, Lipoprotein and Atherosclerosis Analysis Laboratory.

Histological Analysis

The liver tissue samples were fixed in 10% neutral formalin (Sigma-Aldrich) for 12 h and then stored in 70% (v/v) ethanol until processed for histology. Samples were embedded in paraffin and processed by Children's Healthcare of Atlanta Pathology Services. Histologic sections (5 µm) were cut and stained with H&E. The liver histology was assessed in a blinded fashion by a certified veterinary pathologist (S.G.). Representative micrographs were collected using the Emory University Integrated Cellular Imaging Core.

Bile Acid Measurements

In order to characterize bile acid metabolism in the ASBT knockout-first mice, feces were collected from single-housed adult male and female mice over a 72-hour period. The total fecal bile acid content was measured by enzymatic assay (2, 10). Non-fasted mice were sacrificed, and small intestine plus luminal contents, liver, and gallbladder were removed en bloc to measure the bile acid pool size. Total bile acids were extracted in ethanol (11), measured by enzymatic assay, and analyzed by HPLC as described (12). Individual bile acid species were identified using an evaporative light scatter detector (Alltech ELSD 800). Bile acids were quantified by comparison to known amounts of authentic standards purchased from Steraloids (Newport, RI). Quantitative analysis of the biliary bile acids from chow or norUDCA-fed mice was carried out at the Clinical Mass Spectrometry Laboratory at Cincinnati Children's Hospital Medical Center. Briefly, biliary bile acids were extracted on a reverse-phase/solid-phase cartridge and analyzed using a Waters Quattro Premier XE triple quadruple mass spectrometer interfaced with Aquity UPLC system. Quantification of bile acids was based on a validated isotope dilution mass spectrometry method (13). Calibration curves were built with 20 of the mouse bile acids using the single ion recording (SIR) function on the mass spectrometer (LLOQ = 5 ng/ml; ULOQ = 1000 ng/ml). The bile acid

Hydrophobic index was calculated according to Heuman using a value of -0.68 for the norUDCA anion (14, 15).

For the norUDCA feeding studies, fecal samples were collected from cages of group-housed mice with standard bedding at the end of the 7-day chow or *nor*UDCA feeding period. The fecal bile acid composition was determined using a Hewlett-Packard Agilent gas chromatography-mass spectrometer (GC/MS) in the Department of Pediatrics Biomarkers Core Facility at Emory University. Briefly, 50 mg of crushed dried feces mixed with 10 nmol of internal standard, 5β-Cholanic acid 7α, 12α-diol (Steraloids), resuspended in 75% methanol plus 250 mM NaOH and heated at 80°C for 2 h. After centrifugation, the bile acids were extracted on a reverse-phase/solid-phase cartridge (C-18 columns; Supelco) and dried down under nitrogen. The bile acids were then methylated by resuspension in acetyl-chloride (Sigma-Aldrich)/methanol (1:20; v:v) and heating at 55ºC for 20 min. For derivatization, the samples were dried under nitrogen, resuspended in N,O-Bis(trimethylsilyl)trifluoroacetamide (Sigma-Aldrich)/ pyridine (Sigma-Aldrich)/ chlorotrimethyl-silane (Sigma-Aldrich) (5:5:0.1; v:v:v), and incubated for 1 h at room temperature. The derivatized bile acids were then dried at room temperature and resuspended in heptane for GC/MS analysis and compared to authentic standards for norUDCA, UDCA, CDCA, LCA, DCA, CA, αMCA, βMCA, and ωMCA (16).

RNA-Seq Analysis

Total RNA was extracted from frozen liver tissue using TRIzol reagent (Invitrogen, Carlsbad, CA). RNA-Seq libraries were prepared by Novogene Co., Ltd and sequenced on an Illumina HiSeq1000 system. The original data obtained from the high throughput sequencing platforms were transformed to sequenced reads by base calling. Raw data are recorded in a FASTQ file which contains sequenced reads and corresponding sequencing quality information. Sequences were aligned to the mouse genome using STAR (17). The number of reads that mapped to each

gene was used to quantify RNA expression, which is indicated as fragments per kilobase or transcript sequence per million base pairs sequenced (18). Differential expression analysis was performed using the DESeq2 R package of Bioconductor (19). The resulting P values were adjusted using the Benjamini-Hochberg procedure to control for the false discovery rate (20). Differentially expressed genes with a fold change > 1.0 and adjusted P < 0.05 were selected for functional annotation (GEO series accession number: GSE145020). Pathway analysis of the RNA-Seq data was performed using MetaCore (GeneGo Inc, Saint Joseph, MN) with a threshold fold change of 2 and P-value of 0.05 to indicate a statistically significant difference.

Measurement of Cl^- currents

Membrane currents were measured by whole-cell patch clamp techniques using mouse large cholangiocytes (MLC) (21, 22). To generate the MLCs, cholangiocytes were isolated from normal mice (BALB/c) and immortalized by transfection with the SV40 large-T antigen gene as described (21). MLC demonstrate properties similar to those of freshly isolated large mouse cholangiocytes and functionally express TMEM16A (23). Coverslips with MLC cells were mounted in the recording chamber and perfused with a standard extracellular solution containing: 140 mM NaCl, 4 mM KCl, 1 mM $CaCl_2$, 2 mM $MgCl_2$, 1 mM KH_2PO4, 10 mM D-glucose, and 10 mM HEPES/NaOH (pH 7.4). The patch clamp studies were conducted in the standard whole-cell configuration at room temperature using an Axopatch 200B amplifier (Axon instruments). The patch pipettes were pulled from Warner instrument glass (Model G120F-3) and had a resistance of 4–5 MΩ after filling with the standard intracellular solution containing: 130 mM KCl, 10 mM NaCl, 2 mM $MgCl_2$, 10 mM HEPES/KOH, 0.5 mM $CaCl_2$, and 1 mM EGTA (pH 7.3), corresponding to a free $[Ca^{2+}]_i$ of ~100 nM. The free $[Ca^{2+}]_i$ was calculated using software kindly provided by Dr. Guy Droogmans (KU Leuven, Leuven Belgium). Recordings were filtered at 1 kHz and sampled at 10 kHz for storage on a computer and analyzed using pCLAMP 11.0.3 software (Molecular Devices). Results are

compared with control studies measured on the same day to minimize any effects of day-to-day variability and reported as current density (pA/pF) to normalize for differences in cell size (23, 24).

TMEM16A silencing

TMEM16A siRNA (TMEM16A-HSS123904) was used to inhibit TMEM16A expression in whole-cell patch clamp experiments. The following sequences of 25-nucleotide long siRNAs were designed and synthesized by Ambion (ThermoFisher Scientific, Cat# 4390827) and transfected in MLCs using Lipofetamine 3000; antisense: UUCUAUAGAUGAUAACUCCdGdA and sense: GGAGUUAUCAUCUAUAGAAdTdT. Negative control from OriGene (SR 30004) was used in control transfections. Block-it TM Fluorescent Oligo (Catalog #2013, Invitrogen) was used to optimize transfection conditions and select transfected cells for whole-cell patch clamp current recording. Experiments were performed 48-72 hours after transfection and the degree of TMEM16A silencing was evaluated by Western Blot analysis. Briefly, protein was extracted from MLCs with RIPA buffer containing protease inhibitors (Roche cOmplete Protease Inhibitor cocktail). Following protein normalization (ThermoFisher, BCA Protein Assay, #23223) 25 μg of protein per lane was separated on a 4%–15% gels (Bio-Rad) and transferred to a PVDF membrane (Bio-Rad) at 70V for 90 minutes. Membranes were blocked in 5% nonfat dry milk before probing with rabbit anti-mouse TMEM16A antibody (1:1000, Alomone Laboratories, Cat# ACL-011) overnight at 4°C. The following day, the membrane was incubated for 1 hour at room temperature in HRP-conjugated secondary antibody Rabbit IgG (1:10,000, Jackson Immuno Research Laboratories, Cat# 111-035-144). Signal was visualized using Amersham ECL Western Blotting Detection Kit (GE Healthcare, Cat# RPN2209).

Luciferase Assay

The Huh7 cells were seeded in a 24-well plate at 1 X 10^5 cells/well in monolayers and cultured at 37°C and 5% CO_2 in modified Eagle's minimal essential medium (EMEM) supplemented with 10%

fetal bovine serum (FBS) and 1% penicillin-streptomycin. The following day, cells at ~85% confluency were transfected using Lipofectamine3000 (Invitrogen) to introduce a control renilla expression plasmid (0.005 µg), where the renilla luciferase gene is driven by a constitutively active promoter from the HSV thymidine kinase gene to monitor transfection efficiency, along with the following plasmid combinations: 1) a chimeric nuclear receptor encoding the ligand binding domain of mouse PXR fused to the DNA binding domain of GAL4 (pGAL4-mPXR-LBD, 0.25 µg) and a 5x Upstream Activation Sequence (UAS)-luciferase reporter (pFR-Luc, 0.025 µg), 2) expression plasmids for human FXR (0.025 µg), RXRα (0.025 µg), and an FXR-responsive luciferase reporter (pECRE-Luc, 0.25 µg), or 3) expression plasmids for human VDR (0.025 µg), RXRα (0.025 µg), and a VDR-responsive luciferase reporter (hCYP24-luc, 0.25 µg). The next day, triplicate wells were incubated in charcoal stripped 10% FBS-containing medium plus vehicle (DMSO), different concentrations of norUDCA, or different concentrations of the following positive control ligands: 5-Pregnen-3β-ol-20-one-16α-carbonitrile (pregnenolone 16α-carbonitrile, a mouse PXR ligand was obtained from Sigma-Aldrich), 3-[2-[2-chloro-4-[[3-(2,6-dichlorophenyl)-5-(1-methylethyl)-4-isoxazolyl]methoxy]phenyl]ethenyl]-benzoic acid (GW4064, an FXR ligand was obtained from Cayman Chemical), or 25-hydroxy-vitamin D3 (a vitamin D receptor ligand was obtained from Sigma-Aldrich). After a 24-hour treatment, cells were washed in 1X PBS, harvested and processed using Luciferase assay reagents (Dual-Luciferase Reporter Assay System) from Promega. The bioluminescence was monitored using a luminometer (Synergy HTX, Biotek). The results were normalized to TK-renilla activity and presented as relative fold change over the DMSO vehicle group.

In vitro Colesevelam Bile Acid Binding Assay

Bile acid binding to colesevelam was carried out as described (25). Briefly, bile acids were dissolved in simulated intestinal fluid (50 mM KH_2PO_4, 22.4 mM NaOH, pH 6.8) at a concentration

of 2 mM for TDCA or 6 mM for GCDA and norUDCA, and pre-incubated for 30 min on a rotator at 37°C prior to addition to 7.5 mg of colesevelam hydrochloride. Samples were withdrawn at 0, 0.5, 1, 2, 3, 4 and 5 h, filtered through a 0.2 μm syringe filter, and the filtrate was analyzed by enzymatic assay to determine the free bile acid concentration. The difference between the initial (0 h) and free bile acid concentrations was used to calculate the amount of the bile acid bound per mg of colesevelam hydrochloride.

Statistics

For the box and whisker plots, median values (line), interquartile range (boxes), and min to max values (whiskers) are shown. For the liver BA composition analysis and gene expression, mean value ± SD is shown. The data were evaluated for statistically significant differences using the Mann-Whitney test, the two-tailed Student's t test, ANOVA (with a Tukey-Kramer honestly significant difference post-hoc test) or Sidak's multiple comparisons test (GraphPad Prism; Mountain View, CA). Values with different superscript letters are significantly different ($P < 0.05$).

Study approval

All animal experiments were approved by the Emory University Institutional Animal Care and Use Committee in accordance with NIH guidelines for the ethical treatment of animals.

References

1. Schneider Aguirre R, and Karpen SJ. Inflammatory mediators increase SUMOylation of retinoid X receptor alpha in a c-Jun N-terminal kinase-dependent manner in human hepatocellular carcinoma cells. *Mol Pharmacol.* 2013;84(2):218-26.
2. Dawson PA, Haywood J, Craddock AL, Wilson M, Tietjen M, Kluckman K, et al. Targeted deletion of the ileal bile acid transporter eliminates enterohepatic cycling of bile acids in mice. *J Biol Chem.* 2003;278(36):33920-7.
3. Ferrebee CB, Li J, Haywood J, Pachura K, Robinson BS, Hinrichs BH, et al. Organic Solute Transporter α-β Protects Ileal Enterocytes From Bile Acid–Induced Injury. *Cellular and Molecular Gastroenterology and Hepatology.* 2018;5(4):499-522.
4. Halilbasic E, Fiorotto R, Fickert P, Marschall HU, Moustafa T, Spirli C, et al. Side chain structure determines unique physiologic and therapeutic properties of norursodeoxycholic acid in Mdr2-/- mice. *Hepatology.* 2009;49(6):1972-81.
5. Rao A, Kosters A, Mells JE, Zhang W, Setchell KD, Amanso AM, et al. Inhibition of ileal bile acid uptake protects against nonalcoholic fatty liver disease in high-fat diet-fed mice. *Science translational medicine.* 2016;8(357):357ra122.
6. Fuchs CD, Paumgartner G, Mlitz V, Kunczer V, Halilbasic E, Leditznig N, et al. Colesevelam attenuates cholestatic liver and bile duct injury in Mdr2(-/-) mice by modulating composition, signalling and excretion of faecal bile acids. *Gut.* 2018;67(9):1683-91.
7. Fickert P, Wagner M, Marschall HU, Fuchsbichler A, Zollner G, Tsybrovskyy O, et al. 24-norUrsodeoxycholic acid is superior to ursodeoxycholic acid in the treatment of sclerosing cholangitis in Mdr2 (Abcb4) knockout mice. *Gastroenterology.* 2006;130(2):465-81.
8. Tian J, Brown LA, Jones DP, Levin MS, Wang L, Rubin DC, et al. Intestinal redox status of major intracellular thiols in a rat model of chronic alcohol consumption. *JPEN J Parenter Enteral Nutr.* 2009;33(6):662-8.
9. Temel RE, Lee RG, Kelley KL, Davis MA, Shah R, Sawyer JK, et al. Intestinal cholesterol absorption is substantially reduced in mice deficient in both ABCA1 and ACAT2. *J Lipid Res.* 2005;46(11):2423-31.
10. Beuling E, Kerkhof IM, Nicksa GA, Giuffrida MJ, Haywood J, aan de Kerk DJ, et al. Conditional Gata4 deletion in mice induces bile acid absorption in the proximal small intestine. *Gut.* 2010;59(7):888-95.
11. Schwarz M, Russell DW, Dietschy JM, and Turley SD. Marked reduction in bile acid synthesis in cholesterol 7alpha-hydroxylase-deficient mice does not lead to diminished tissue cholesterol turnover or to hypercholesterolemia. *J Lipid Res.* 1998;39(9):1833-43.
12. Torchia EC, Labonte ED, and Agellon LB. Separation and quantitation of bile acids using an isocratic solvent system for high performance liquid chromatography coupled to an evaporative light scattering detector. *Anal Biochem.* 2001;298(2):293-8.
13. Hagio M, Matsumoto M, Fukushima M, Hara H, and Ishizuka S. Improved analysis of bile acids in tissues and intestinal contents of rats using LC/ESI-MS. *Journal of lipid research.* 2009;50(1):173-80.
14. Heuman DM. Quantitative estimation of the hydrophilic-hydrophobic balance of mixed bile salt solutions. *J Lipid Res.* 1989;30(5):719-30.
15. Stravitz RT, Hylemon PB, Heuman DM, Hagey LR, Schteingart CD, Ton-Nu HT, et al. Transcriptional regulation of cholesterol 7 alpha-hydroxylase mRNA by conjugated bile acids in primary cultures of rat hepatocytes. *J Biol Chem.* 1993;268(19):13987-93.
16. Rao A, van de Peppel IP, Gumber S, Karpen SJ, and Dawson PA. Attenuation of the Hepatoprotective Effects of Ileal Apical Sodium Dependent Bile Acid Transporter (ASBT)

Inhibition in Choline-Deficient L-Amino Acid-Defined (CDAA) Diet-Fed Mice. *Front Med (Lausanne).* 2020;7:60.
17. Dobin A, and Gingeras TR. Mapping RNA-seq Reads with STAR. *Curr Protoc Bioinformatics.* 2015;51:11 4 1- 4 9.
18. Anders S, Pyl PT, and Huber W. HTSeq--a Python framework to work with high-throughput sequencing data. *Bioinformatics.* 2015;31(2):166-9.
19. Love MI, Huber W, and Anders S. Moderated estimation of fold change and dispersion for RNA-seq data with DESeq2. *Genome Biol.* 2014;15(12):550.
20. Li J, Witten DM, Johnstone IM, and Tibshirani R. Normalization, testing, and false discovery rate estimation for RNA-sequencing data. *Biostatistics.* 2012;13(3):523-38.
21. Ueno Y, Alpini G, Yahagi K, Kanno N, Moritoki Y, Fukushima K, et al. Evaluation of differential gene expression by microarray analysis in small and large cholangiocytes isolated from normal mice. *Liver Int.* 2003;23(6):449-59.
22. Glaser S, Wang M, Ueno Y, Venter J, Wang K, Chen H, et al. Differential transcriptional characteristics of small and large biliary epithelial cells derived from small and large bile ducts. *Am J Physiol Gastrointest Liver Physiol.* 2010;299(3):G769-77.
23. Dutta AK, Khimji AK, Kresge C, Bugde A, Dougherty M, Esser V, et al. Identification and functional characterization of TMEM16A, a Ca2+-activated Cl- channel activated by extracellular nucleotides, in biliary epithelium. *J Biol Chem.* 2011;286(1):766-76.
24. Dutta AK, Khimji AK, Sathe M, Kresge C, Parameswara V, Esser V, et al. Identification and functional characterization of the intermediate-conductance Ca(2+)-activated K(+) channel (IK-1) in biliary epithelium. *Am J Physiol Gastrointest Liver Physiol.* 2009;297(5):G1009-18.
25. Krishnaiah YS, Yang Y, Bykadi S, Sayeed VA, and Khan MA. Comparative evaluation of in vitro efficacy of colesevelam hydrochloride tablets. *Drug Dev Ind Pharm.* 2014;40(9):1173-9.

Table 1. Physical and Permeability Properties of UDCA versus norUDCA

	**UDCA**	**norUDCA**
**Formula**	C24H40O4	C23H38O4
**Molecular Weight**	392.56[1]	378.553[1]
**xLogP3**	4.9[1]	4.6[1]
**Hydrogen Bond Donor**	3[1]	3[1]
**Hydrogen Bond Acceptor**	4[1]	4[1]
**Critical Micelle Concentration (mM)**	7[2]	17[3]
**Apparent Permeability (x 10^4) (cm/sec in perfused rat jejunum)**	0.67[4]	0.64[4]

1. Values computed by PubChem, XLogP3 3.0, Cactvs 3.4.6.11.
2. Hofmann A and Roda A, Hepatology 1984: 25: 1477-1489.
3. Roda A, Hofmann AF, Mysels KJ. J Biol Chem 1983; 258: 6362-6370.
4. Dupas J-L et al, Lipids 2018; 53: 465-468.

Table 2. Plasma Chemistries in Chow and norUDCA Diet-fed Wild Type and *Asbt*$^{-/-}$ Mice

Variable	WT (n)		*Asbt*$^{-/-}$ (n)	
	Chow (6)	norUDCA (7)	Chow (7)	norUDCA (7)
**Albumin (g/dL)**	1.8 ± 0.8^{a}	3.1 ± 0.5^{a,b}	2.6 ± 0.5^{b}	2.9 ± 0.4^{b}
**Alkaline phosphatase (U/L)**	38 ± 12	37 ± 14	33 ± 12	45 ± 5
**Alanine aminotransferase (U/L)**	85 ± 59	61 ± 27	120 ± 49	95 ± 39
**Aspartate aminotransferase (U/L)**	116 ± 67^{a,b}	99 ± 28^{a}	199 ± 70^{b}	146 ± 48^{a,b}
**Cholesterol (mg/dL)**	95 ± 27^{a}	151 ± 30^{a,b}	109 ± 18^{b}	148 ± 41^{b}
**Gamma-glutamyl transpeptidase (U/L)**	9.6 ± 4.6^{a}	15.4 ± 4.9^{a,b}	15.4 ± 2.8^{a,b}	17.1 ± 3.8^{b}
**Total bilirubin (mg/dL)**	3.0 ± 1.3^{a,b}	2.6 ± 0.9^{a}	3.6 ± 0.6^{b}	2.8 ± 0.7^{a,b}
**Total protein (g/dL)**	3.6 ± 0.6	4.6 ± 0.9	3.6 ± 0.9	4.6 ± 0.5

Values are expressed as the mean ± SD. The number of mice per group are indicated (n). Values with different superscript letters are significantly different ($P < 0.05$) by ordinary two-way ANOVA and Sidak's multiple comparisons test.

Table 3. Plasma Chemistries in Wild Type Mice after co-administration of norUDCA with an ASBTi or Colesevelam

Variable	Control	ASBTi	Colesevelam	*nor*UDCA	*nor*UDCA ASBTi	*nor*UDCA Colesevelam
**Albumin (g/dL)**	3.3 ± 0.2	3.0 ± 0.2	3.1 ± 0.2	3.4 ± 0.2	3.0 ± 0.7	3.5 ± 0.2
**ALP (U/L)**	6.4 ± 6.1	1.6 ± 2.2	8.8 ± 7.2	6.4 ± 5.4	4.0 ± 2.8	6.4 ± 4.6
**ALT (U/L)**	96 ± 27	96 ± 27	118 ± 46	74 ± 23	143 ± 96	97 ± 53
**AST (U/L)**	176 ± 60	170 ± 46	247 ± 145	90 ± 25	193 ± 141	115 ± 33
**CHOL (mg/dL)**	123 ± 13[a]	125 ± 10[a]	110 ± 9[a]	171 ± 14[b]	130 ± 44[a,b]	148 ± 10[a,b]
**GGT (U/L)**	19.2 ± 4.4	16.8 ± 3.3	14.4 ± 3.6	18.4 ± 6.1	15.2 ± 3.3	16.8 ± 3.3
**TBILI (mg/dL)**	2.6 ± 0.3[a]	3.4 ± 0.4[a]	2.6 ± 0.5[a]	2.0 ± 0.4[b]	3.1 ± 1.0[a]	1.9 ± 0.4[b]
**TP (g/dL)**	4.8 ± 0.3	4.3 ± 0.3	4.7 ± 0.4	5.1 ± 0.4	4.4 ± 1.2	5.4 ± 0.4

Values are expressed as the mean ± SD (n = 5 mice per group). Values with different superscript letters are significantly different ($P < 0.05$) by ordinary two-way ANOVA and Sidak's multiple comparisons test. ALP, alkaline phosphatase; ALT, alanine aminotransferase; AST, aspartate aminotransferase; CHOL, cholesterol; GGT, gamma-glutamyl transpeptidase; TBILI, total bilirubin; TP, total protein.

Supplemental Figures and Legends

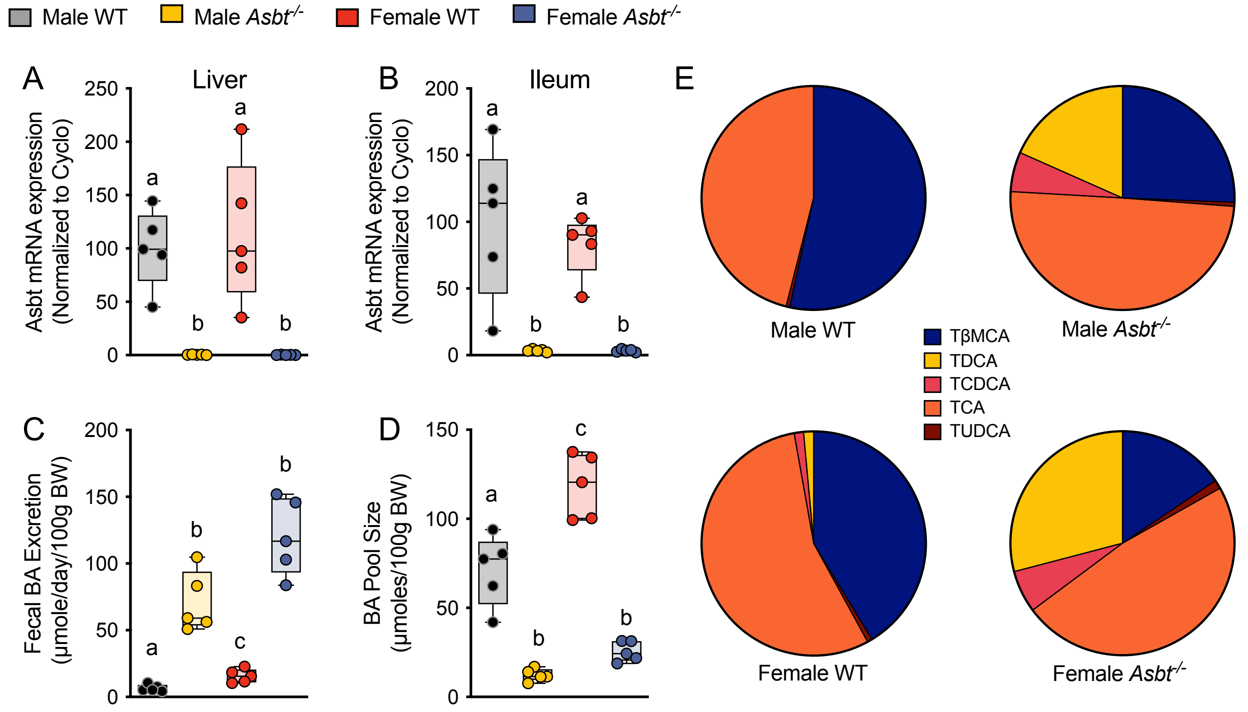

Supplemental Figure 1. ASBT expression, bile acid excretion, bile acid pool size, and pool composition in wild type and ASBT knockout-first (*Asbt*$^{-/-}$) mice. (**A**) ASBT mRNA levels in liver and (**B**) ileum of male and female WT and *Asbt*$^{-/-}$ mice. RNA was isolated from individual mice and used for real-time PCR analysis. The mRNA expression was normalized to cyclophilin and are shown relative to male WT mice (set at 100%) for each tissue. (**C**) Fecal bile acid excretion in male and female WT and *Asbt*$^{-/-}$ mice. The data are expressed as µmoles/per day per 100 g body weight. (**D**) Bile acid pool size in in male and female WT and *Asbt*$^{-/-}$ mice. The data are expressed as µmoles per 100 g body weight. (A) through (D) Median values (line), interquartile range (boxes), and min to max values (whiskers) are shown (n = 5 mice per group). The data were evaluated for statistically significant differences using an ordinary one-way ANOVA with a Tukey's multiple comparisons test. Values with different superscript letters are significantly different ($P < 0.05$). (**E**) Bile acid pool composition in male and female WT and *Asbt*$^{-/-}$ mice. Pie charts show the mean values (n = 5 mice per group) for the percent compositions of the indicated bile acids. All mice were 3 months of age.

ASBT mRNA expression is reduced to background levels in liver and ileum of *Asbt*$^{-/-}$ mice. Fecal bile acid excretion was increased by approximately 8 to 10-fold in male and female *Asbt*$^{-/-}$ versus WT mice. The increased fecal bile acid excretion was associated with approximately 80 percent reductions in the bile acid pool size of male and female *Asbt*$^{-/-}$ mice. Analysis of the bile acid pool composition by HPLC revealed a large reduction in the proportion of 6-hydroxylated bile acid species and concomitant increase in the proportion of taurocholic acid and its gut microbiota-derived product taurodeoxycholic acid. These changes in fecal bile acid excretion, bile acid pool size and composition in the C57BL/6NJ ASBT knockout-first mice recapitulates the major bile acid phenotype of the previously characterized Asbt knockout mouse model (2). BA, bile acid; BW, body weight; TβMCA, tauro-beta-muricholic acid; TCA, taurocholic acid; TCDCA, taurochenodeoxycholic acid; TDCA, taurodeoxycholic acid; TUDCA, tauroursodeoxycholic acid; WT, wild type.

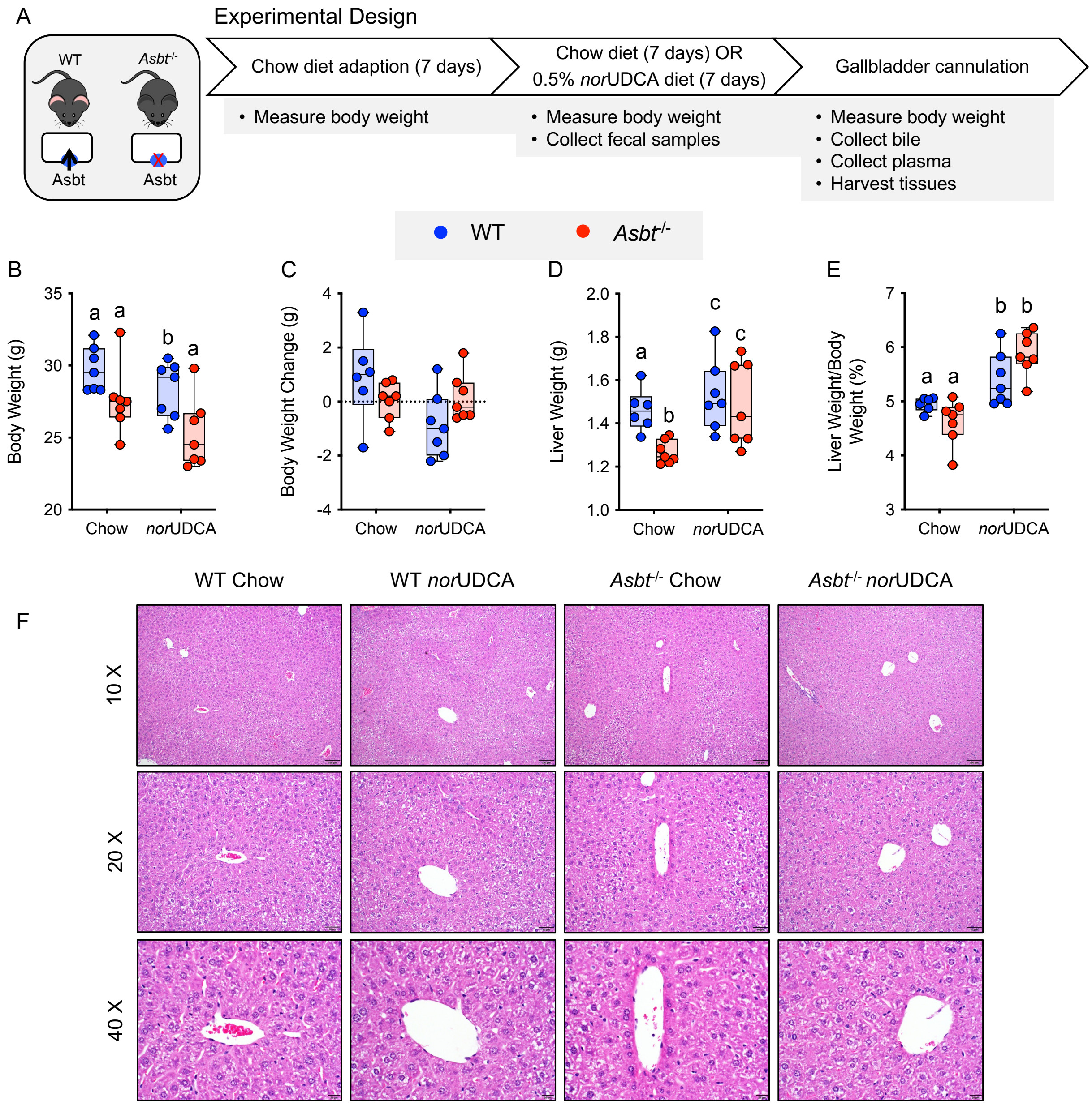

Supplemental Figure 2. Experimental scheme and morphological response to norUDCA treatment in WT and *Asbt*$^{-/-}$ mice. (**A**) Experimental design. (**B**) Body weight after 7-days of feeding chow or chow plus 0.5% norUDCA. (**C**) Body weight change after 7-days of feeding chow or chow plus 0.5% norUDCA. (**D**) Liver weight. (**E**) Liver to body weight ratio. Median values (line), interquartile range (boxes), and min to max values (whiskers) are shown, n = 6-7 mice per group. The data were evaluated for statistically significant differences using an ordinary two-way ANOVA with a Tukey's multiple comparisons test. Distinct lowercase letters indicate significant differences between groups ($P < 0.05$). (**F**) Hematoxylin and eosin-stained liver sections (original magnification 10X, 20X, and 40X) from the indicated genotypes and treatments groups. Scale bars, 200 µm, 100 µm, 50 µm at 10X, 20X, and 40X, respectively).

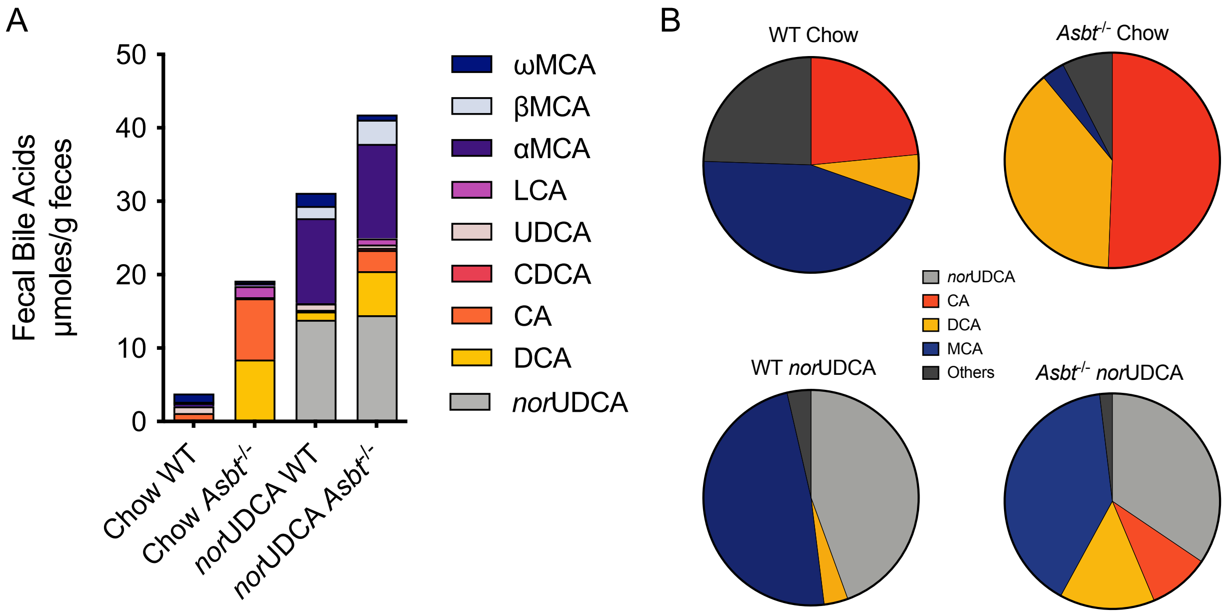

Supplemental Figure 3. Effect of norUDCA treatment on the fecal bile acid profile in WT and *Asbt*$^{-/-}$ mice. (**A**) norUDCA treatment increased the excretion of endogenous bile acids and the amount of 6-hydroxylated bile acids in feces. (**B**) Pie charts for the fecal bile acid profiles. Mean values are shown for fecal samples collected from cages of group-housed mice (n = 6-7 mice per genotype and treatment condition).

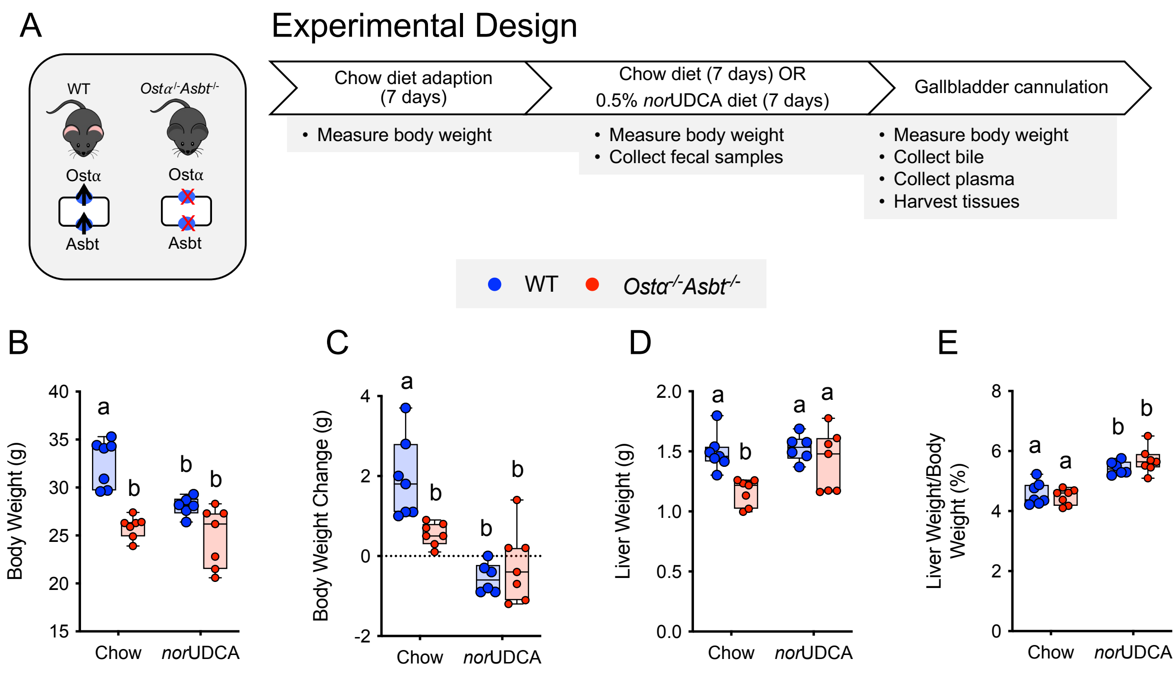

Supplemental Figure 4. Experimental scheme and morphological response to *nor*UDCA treatment in WT and *Osta*$^{-/-}$*Asbt*$^{-/-}$ mice. (**A**) Experimental design. (**B**) Body weight after 7-days of feeding chow or chow plus 0.5% *nor*UDCA. (**C**) Body weight change after 7-days of feeding chow or chow plus 0.5% norUDCA. (**D**) Liver weight. (**E**) Liver to body weight ratio. Median values (line), interquartile range (boxes), and min to max values (whiskers) are shown, n = 5-7 mice per group. The data were evaluated for statistically significant differences using an ordinary two-way ANOVA with a Tukey's multiple comparisons Distinct lowercase letters indicate significant differences between groups (P < 0.05).

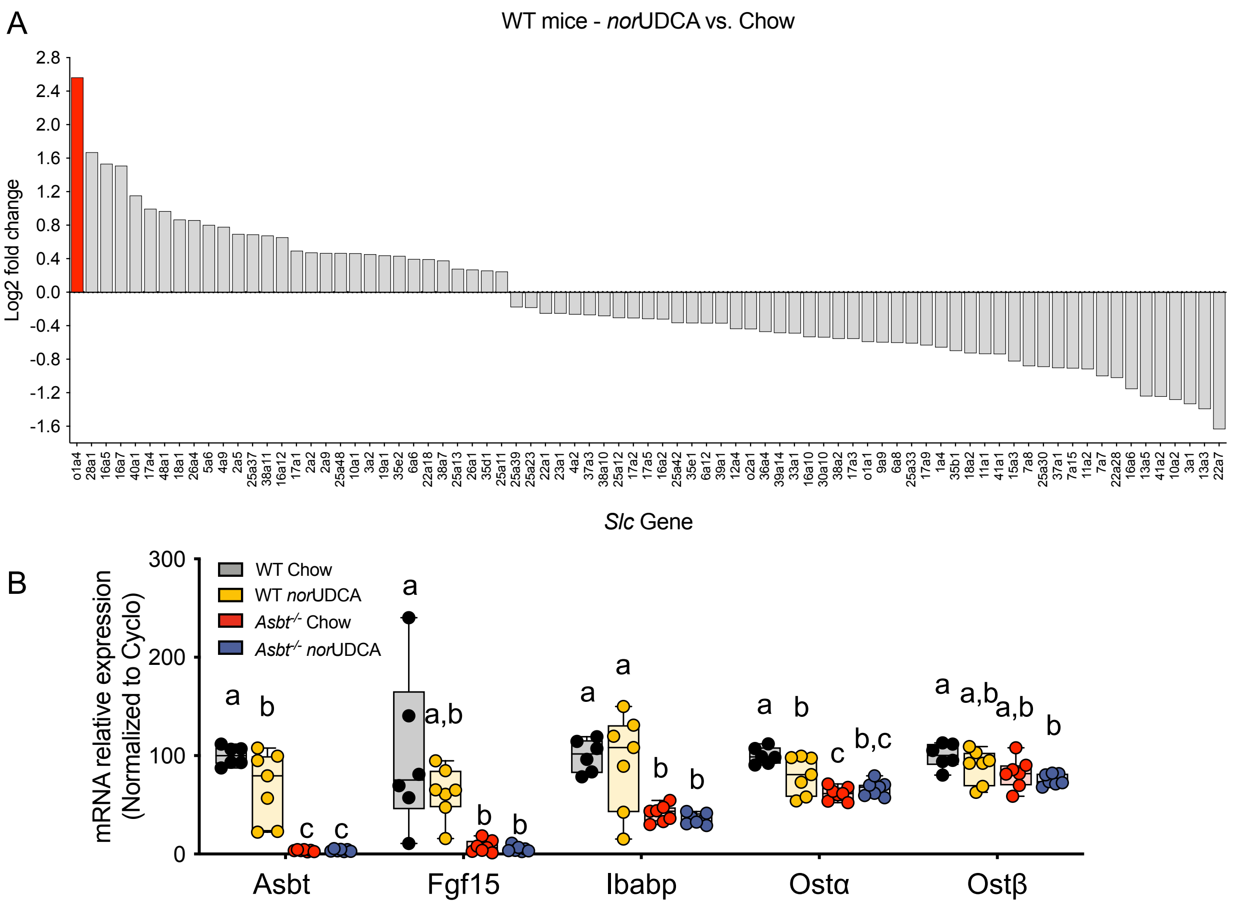

Supplemental Figure 5. Effect of norUDCA treatment on hepatic and ileal gene expression. (**A**) RNA-Seq analysis of livers from WT mice fed chow or the norUDCA-containing diet. Differentially expressed *SLC* membrane transporter genes (P < 0.05; n = 6 mice per group) in the norUDCA-treated versus chow mice are shown. (**B**) Ileal expression of the indicated bile acid-related genes in WT and *Asbt*$^{-/-}$ mice fed chow or the norUDCA-containing diet for 7 days. RNA was isolated from ileum of individual mice and used for real-time PCR analysis. The mRNA expression was normalized using cyclophilin and the results for each gene are expressed relative to chow-fed WT mice. Median values (line), interquartile range (boxes), and min to max values (whiskers) are shown, n = 6-7 mice per group. The data were evaluated for statistically significant differences using an ordinary one-way ANOVA with a Tukey's multiple comparisons test. Distinct lowercase letters indicate significant differences between groups (P < 0.05).

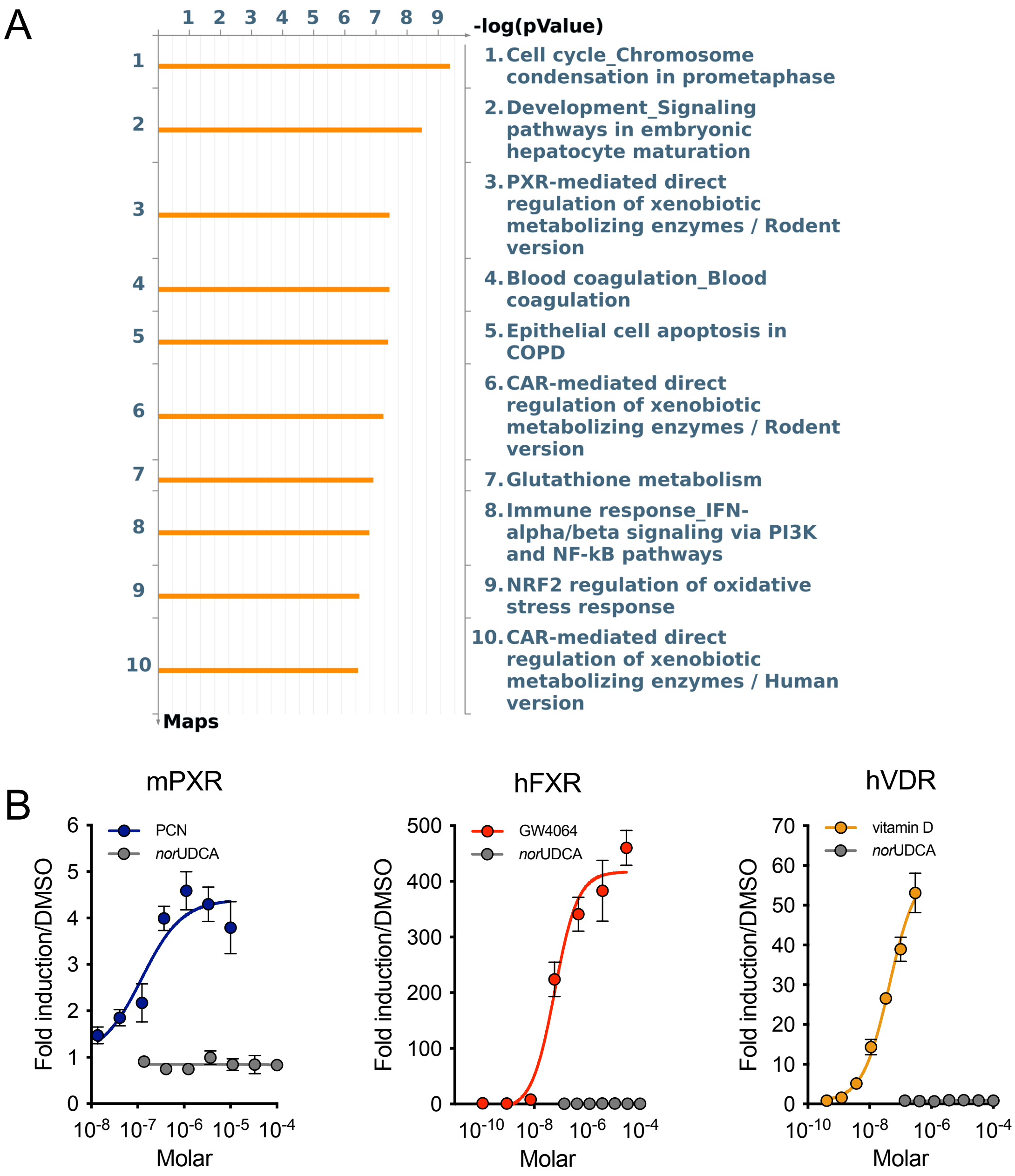

Supplemental Figure 6. Candidate pathways regulated in mouse liver by norUDCA. (**A**) Pathway analysis of liver gene expression changes in norUDCA treated versus chow-fed mice using a threshold fold change of 2 and *P* value of 0.05. The top 10 pathways and -log(pValue) are shown. (**B**) norUDCA does not directly activate mouse PXR, human FXR or human VDR in transfected Huh7 cells. Huh7 cells were transfected with the indicated nuclear receptor expression and reporter plasmid combinations. One day following transfection, the cells were treated with the indicated concentrations of positive control ligand or norUDCA for 24 h prior to luciferase activity measurement. The results were normalized to TK-renilla activity and presented as relative fold change versus vehicle (DMSO). Mean values + SD are shown. The experiment was independently conducted at least 3 times for each of the nuclear receptors.

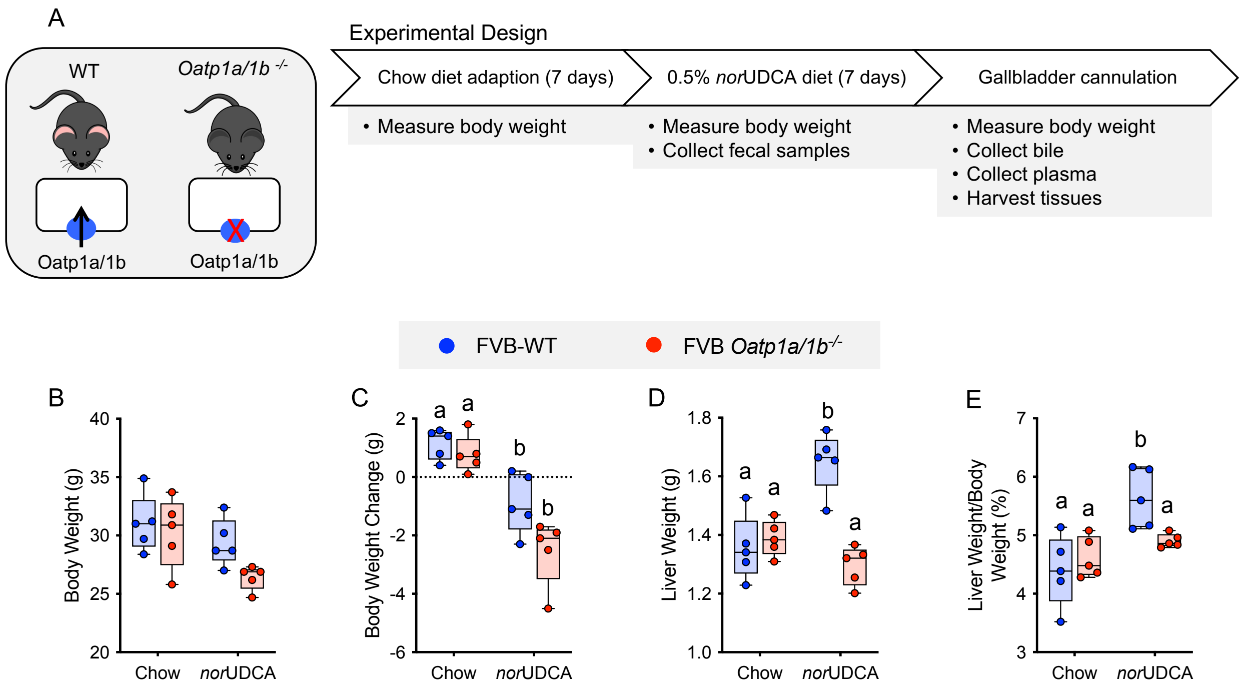

Supplemental Figure 7. Experimental scheme and morphological response to norUDCA treatment in WT and *Oatp1a/1b*$^{-/-}$ mice. (**A**) Experimental design. (**B**) Body weight. (**C**) Body weight change. (**D**) Liver weight. (**E**) Liver to body weight ratio. Median values (line), interquartile range (boxes), and min to max values (whiskers) are shown; n = 5 mice per group. The data were evaluated for statistically significant differences using an ordinary two-way ANOVA with a Tukey's multiple comparisons test. Distinct lowercase letters indicate significant differences between groups ($P < 0.05$).

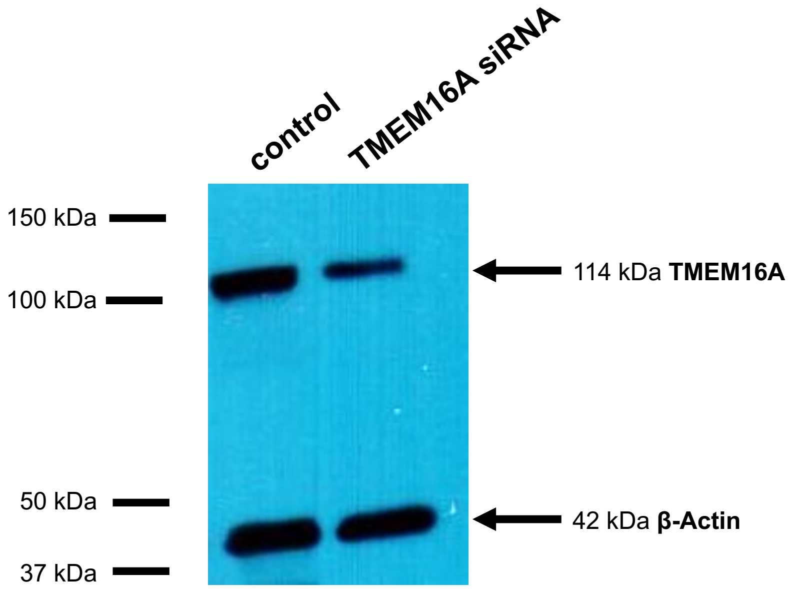

Supplemental Figure 8. Knockdown of TMEM16A expression in Mouse Large Cholangiocytes (MLCs) used for norUDCA treatment and patch clamping studies. Representative western blot demonstrating reduction in TMEM16A protein levels in MLCs following transfection with TMEM16A siRNA as compared to transfection with a nontargeting siRNA (control). Western blotting for β-actin was used as loading control.

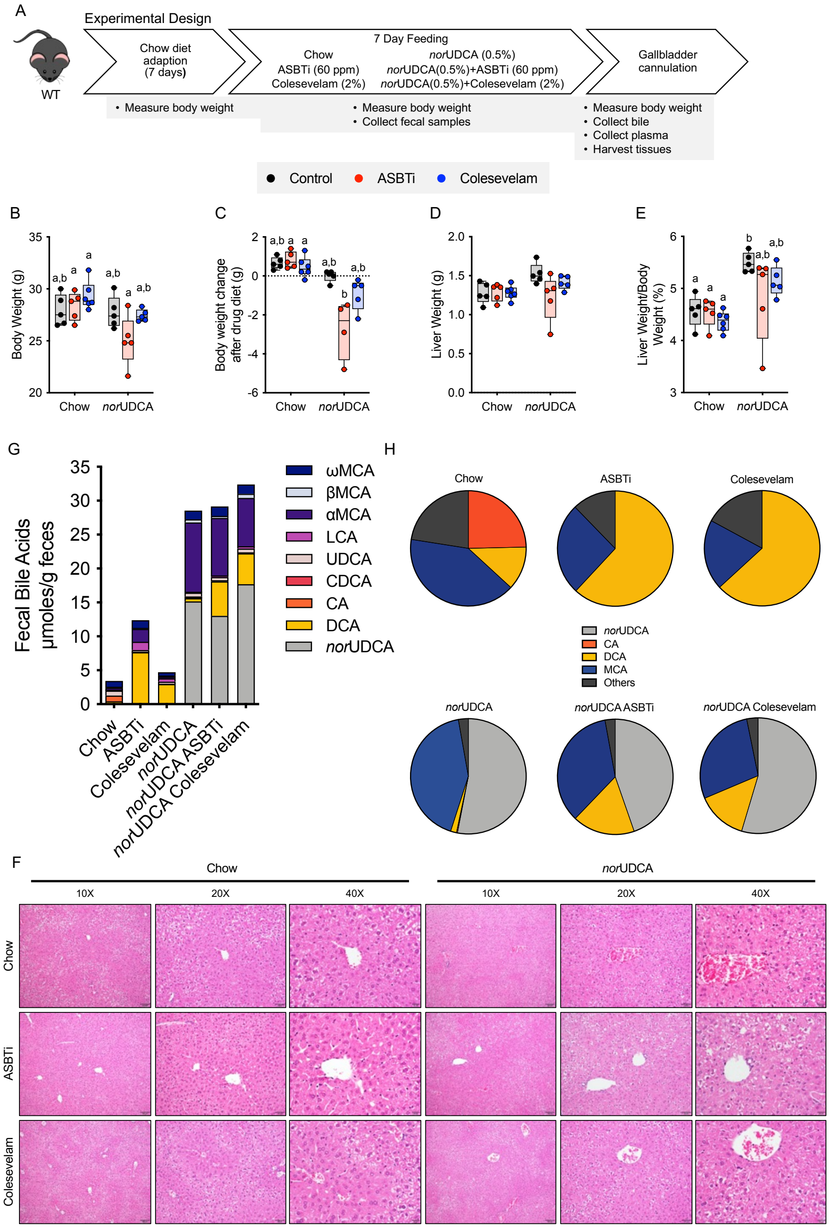

Supplemental Figure 9. Experimental scheme and morphological response of WT mice to co-administration of norUDCA with an ASBT inhibitor or Colesevelam. (**A**) Experimental design. (**B**) Body weight. (**C**) Body weight change. (**D**) Liver weight. (**E**) Liver to body weight ratio. Median values (line), interquartile range (boxes), and min to max values (whiskers) are shown, n = 5 mice per group. The data were evaluated for statistically significant differences using an ordinary two-way ANOVA with a Tukey's multiple comparisons test. Distinct lowercase letters indicate significant differences between groups ($P < 0.05$). (**F**) Hematoxylin and eosin-stained liver sections (original magnification 10X, 20X, or 40X) from the indicated treatments groups. Scale bars, 200 µm, 100 µm, 50 µm at 10X, 20X, and 40X, respectively). (**G**) norUDCA treatment increased the excretion of endogenous bile acids and the amount of 6-hydroxylated bile acids in feces. (**H**) Pie charts for the fecal bile acid profiles. Mean values are shown for fecal samples collected from cages of group-housed mice (n = 5 mice per treatment condition).

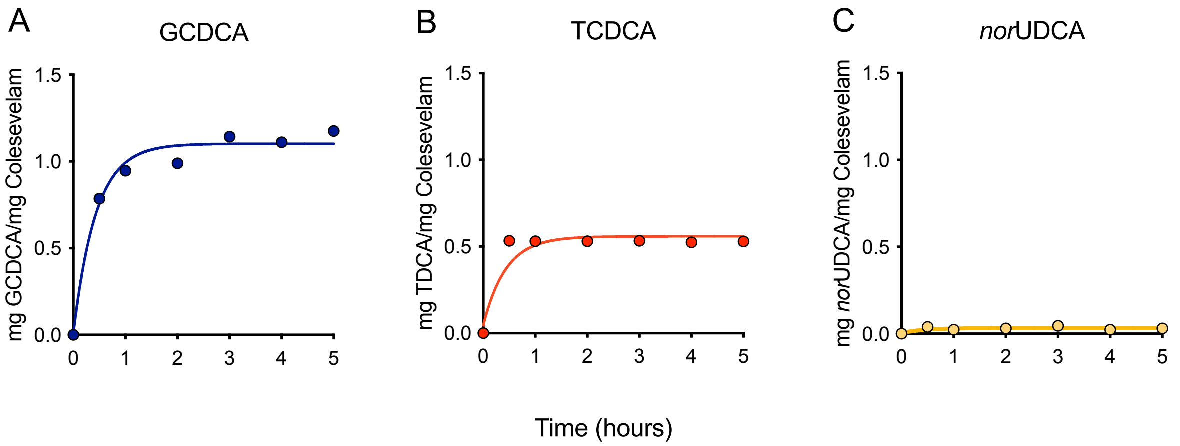

Supplemental Figure 10. In vitro binding of bile acids and norUDCA to colesevelam. (**A**) Glycochenodeoxycholic acid (GCDCA), (**B**) Taurochenodeoxycholic acid TCDCA), (**C**) norUDCA. Bile acids were dissolved in simulated intestinal fluid at a concentration of 2 mM for TCDCA or 6 mM for GCDA and *nor*UDCA and incubated with 7.5 mg of colesevelam hydrochloride at 37°C. Samples were withdrawn at 0, 0.5, 1, 2, 3, 4 and 5 h, filtered through a 0.2 µm syringe filter, and the filtrate was analyzed by enzymatic assay to determine the free bile acid concentration by enzymatic assay. The difference between the initial (0 h) and unbound bile acid concentrations at each time point was used to calculate the amount of the bile acid bound per mg of colesevelam hydrochloride (n = 1 determination per time point). The binding experiments were independently conducted 3 times.
